# Brood reduction caused by sibling cannibalism in *Isodontia harmandi* (Hymenoptera: Sphecidae), a solitary wasp species building communal brood cells

**DOI:** 10.1371/journal.pone.0267958

**Published:** 2022-05-18

**Authors:** Yui Imasaki, Tomoji Endo

**Affiliations:** Department of Biosphere Sciences, School of Human Sciences, Kobe College, Nishinomiya, Hyogo, Japan; Universidade de São paulo, BRAZIL

## Abstract

Sibling rivalry or brood reduction prevailing within bird nests is effectively avoided in solitary aculeate nests because the larvae of wasps and bees usually develop in each brood cell. However, a solitary wasp species, *Isodontia harmandi*, allows us to study brood reduction in a communal brood cell, where up to a dozen larvae develop in a group relying on prey provisioned by a female wasp. To demonstrate brood reduction in this species, we collected nests at various developmental brood stages from fields for five years (2010–2015). There was a significant decrease in the brood size between the nests sampled at the egg or hatchling stages and those at later stages when analyzing only data excluding nests that were parasitized, attacked by predators, or containing deteriorated prey. In whole brood-rearing experiments, we also confirmed that brood reduction occurred in 30 of 39 nests during larval stages and in 23 nests after cocoon stage. Larval survival was affected positively by total prey weight and negatively by brood size, though cocoon survival was not affected. A third-quarter (76%) of larval death was identified as sibling cannibalism through observation by time-lapse recording on multi-larvae rearing experiments. Therefore, we conclude that brood reduction routinely occurs as a result of sibling cannibalism in *I*. *harmandi*. Additionally, as we could not detect any positive effects of clutch size on the amount of provision, female wasps might overproduce offspring due to the unpredictability of available prey resources. Differences in brood size and reduction among sex categories were undetected, except for parental provisions. Thus, sibling cannibalism may efficiently regulate brood size in communal brood cells under prey shortage.

## Introduction

In species where parents provide food and other forms of care for their offspring in a nursery (any confined space) [1], parents tend to produce more offspring than they can rear [2]. This problem of parental overproduction in a nursery is often solved by an intuitively paradoxical phenomenon: brood reduction through various forms of fatal sibling rivalry, such as abortion, obligate or facultative siblicide, filial cannibalism, and sibling cannibalism [1, 3]. Most studies on brood reduction have been conducted in altricial birds (typical nursery animals) [1, 4].

However, brood reduction ubiquitously occurs among diverse plant and animal taxa [1, 3]. Therefore, our understanding of brood reduction should be widened by studying non-avian species [5]. Cannibalism during brood reduction is rarely seen in birds [1, 6]; however, it is relatively common among insects and amphibians [7, 8], but it remains to be uncovered whether sibling cannibalism plays a vital role in brood reduction among these taxa. In insects, there are many examples showing evidence that cannibalism occurs during brood reduction. For example, filial cannibalism plays a role in adjusting brood size to available resources in burying beetle (*Nicrophorus vespilloides*) [9] and passalid beetle (*Cylindrocaulus patalis*) [10]. Both infertile (trophic) and fertile eggs are consumed by sibling larvae emerging earlier in the same clutch of ladybirds (*Harmonia axyridis)* [11] and sub-social stink bugs (*Adomerus triguttulus*) [12]. However, these cases are not discussed in the context of brood reduction through sibling rivalry in the nursery system because their egg clusters are deposited in open space and can be also consumed by other conspecific individuals [13]. In the case of European earwig (*Forficula auricularia*), siblicide and cannibalism may co-occur in a nest, but cannibalism among siblings tends to be avoided [14, 15]. To the best of our knowledge, there is no case where brood reduction routinely occurs by sibling cannibalism among organisms growing together in the nursery system.

Hymenopteran insects, especially aculeate bees and wasps, are sometimes compared with birds because they construct elaborate nests as nurseries and usually lay multiple eggs in the nest, providing food for offspring until independence. Similar patterns of parental care may impose selection favoring parallel evolution of reproductive strategies, such as intraspecific brood parasitism [16, 17] and cooperative breeding [18] in both groups. However, sibling rivalry or brood reduction prevailing within bird nests are effectively avoided in the nests of solitary bees and wasps because their larva usually develops in a “private” brood cell [19]. In eusocial species sibling cannibalism often occurs in the form of egg-eating by ant larva [20] or adult worker of honeybees or polistine wasps in a colony [21], where larva or adult is accessible to its young immobile sibling (i.e., egg) in the colony. In the nest, especially multiple-celled nests built by solitary bees and wasps, neither behavioral interaction nor direct competition occurs between siblings growing in neighboring partitioned cells and there are usually no differently aged siblings in the nest. In the nest of solitary wasps, whose larvae are potentially aggressive and cannibalistic, larval cannibalism occurs only if the larvae encounter each other due to accidental partition break [22]. However, a wasp species, *Isodontia harmandi*, allows us to study brood reduction in a non-avian taxon with a nursery system and costly food provisioning. This wasp species has two unique aspects of nesting biology: “communal brood cell” and “single-sex brood.” The former means that several larvae grow together, sharing a large amount of prey provisioned in a large single cell made by the female wasp. The latter indicates that all larvae grown from a nest are usually females or males [22–25].

Interestingly, behavioral tolerance was observed among the larvae within a nest of *I*. *harmandi*, while larval cannibalism was also reported [22, 23]. Initially, larval cannibalism had been observed in this species, which should be “peaceful,” and was perceived as a surprising and anomaly event [22, 23]. Tsuneki [22] suggested that larval cannibalism sometimes occurs because larvae do not yet become adapted to the “communal brood chamber” system in the incipient condition during its evolutionary process. However, he changed his view, such that larval cannibalism may function as a feeding control mechanism [25], probably because he thought that cannibalism occurred too frequently to be an error. Murota [26, 27] also suggested that larval cannibalism in *I*. *harmandi* may be common and caused by food shortages in the nest through rearing experiments using five nests. However, no study has been conducted on a more rigorous examination of larval cannibalism in this species.

There is another advantage of studying sibling rivalry or brood reduction in hymenopteran insects, such as *I*. *harmandi*. The haplodiploid sex-determination system allows one to evaluate the effect of relatedness on sibling rivalry by comparing the degree of brood reduction between sister brood with high relatedness and brother brood with low relatedness. Thus, *I*. *harmandi* is a valuable model for understanding the evolutionary effects of inevitable sibling rivalry. Taking group development of larvae and sibling cannibalism reported in *I*. *harmandi* into account, we hypothesize that larval cannibalism in *I*. *harmandi* is a mechanism driving brood reduction and enhancing the growth and survival of larvae under critical conditions of food shortage. To evaluate the validity of this hypothesis, we first must know to what extent brood reduction occurs in the nest of *I*. *harmandi* under natural conditions. Next, we must determine the relationship between the initial brood size (i.e., the number of eggs laid by a female wasp in a brood cell) and the amount of prey provisioned by the female to clarify whether the larvae confront food shortage or whether female wasps overproduce their offspring in the nests. Further, we must demonstrate that brood reduction results from sibling cannibalism. When these issues are confirmed, we can only proceed to examine the adaptive aspects of our hypothesis. This study demonstrates that brood reduction commonly occurs due to sibling cannibalism in the nest of *I*. *harmandi*, using data of nests collected from fields during 2010–2015.

In most bird species, patterns of partial brood loss have been documented by census data, i.e., repeated counts of brood size in many nests randomly chosen for study [1]. It is, however, challenging to track changes in the numbers of larvae that survived in the brood cell for specific nests of *I*. *harmandi* without having any impact on the inner conditions of the nests. Therefore, instead of tracking particular nests, we collected as many nests as possible from the study area and counted the number of eggs and larvae in the nest in various stages of nesting and larval development. Furthermore, these brood size data obtained from the field should be screened to eliminate nest data with mortality factors other than sibling interactions (i.e., natural enemy or deterioration of prey conditions) because we focus on brood reduction by sibling cannibalism. Finally, we rearranged brood size data along with the temporal progress of developmental stages in the nests collected at various nesting seasons. However, as Mock and Parker [1] noted, census or this nest sample data help depict the occurrence of brood reduction but not the cause of brood loss. Therefore, we confirmed that larval cannibalism was a primary cause of brood reduction in the nests of *I*. *harmandi* by identifying the fate of larvae in a group from the same brood during the developmental period under laboratory conditions.

This study examines the following questions; 1) does brood reduction occur among the nests of *I*. *harmandi*? 2) Does food shortage occur among the nests of *I*. *harmandi*? 3) Are there differences in brood reduction and the degree of parental investment between female and male broods of *I*. *harmandi*? 4) Is brood reduction a consequence of sibling cannibalism?

This study provides rigorous evidence that brood reduction occurs due to sibling cannibalism in the nests of solitary sphecid wasp *I*. *harmandi*. This is the first discovery of brood reduction that routinely occurs as a result of sibling cannibalism. Although sibling rivalry has been widely known in birds, studying completely different taxa with a nursery system provides us with a completely different case of sibling rivalry, which helps us understand sibling rivalry.

## Materials & methods

### Nesting biology of *I*. *harmandi*

Wasps of the genus *Isodontia* use pre-existing cavities in nature as nests and provide orthopteran or blattodean insects as prey for larvae [28–30]. At first, female wasps of *I*. *harmandi* carry a paralyzed prey item into the nest cavity temporally plugged with a small amount of moss before prey hunting [22, 23]. Then, females lay an egg on the first prey at the anterior part of the mesosternum between the frontal coxae. After that, females usually continue to oviposit on every prey item until several hunting and egg-laying activities cease. Females oviposit from a few eggs up to a maximum of 10–11 eggs in a single brood cell [22, 23], though they sporadically lay one or two eggs after several hunting/egg-laying activities [22]. Additionally, when several oviposition is completed, females usually provide numerous prey items and finally close the cavity’s entrance with a closure plug made of densely packed mosses or other plant materials [28]. According to previous reports, the average number of eggs and prey in a brood cell is 5.4 eggs (1–10) and 37.1–42.3 prey (6–85) [22, 23].

Therefore, *I*. *harmandi* is a mass provider in a single-celled nest in which prey hunting precedes oviposition, but is characterized by a distinct sequence of nesting behavior that multiple egg-laying precedes the storage of a large amount of prey. This exceptional nesting behavior of *I*. *harmandi* should force female wasps to decide on the number of eggs to lay before gathering food for their offspring. The situation is almost the same as that of breeding birds.

### Placement and collection of trap nests

Bamboo-cane traps were installed at the study sites in Tamba-Sasayama City and Takarazuka City, Hyogo Prefecture, central Japan, during the nesting seasons in 2010–2013 and 2015 to obtain the nests of *I*. *harmandi*. We shifted the study sites in one or two years to prevent the wasp populations from declining as a result of nest removal. Traps were installed at Minami-yashiro, in 2010 and 2011, Furuichi in 2012, Kusano in 2013 (all in Tamba-Sasayama City), and Minami-yashiro and Kirihata (Takarazuka City) in 2015. As the Kirihata bamboo-cane trap study [31] was conducted to investigate other wasp species before this study, we shifted our study site to about 100 m north from the previous study area. Nesting activities were observed from late July to early September, with the peak activity in early and mid-August yearly.

Each trap was composed of 12 bamboo canes (inner diameter: M-size, about 10 mm and L-size, about 14 mm) [31], which were knitted with vinyl-coated wire in 2010. Since 2011, we have used a slightly different trap in which eight bamboo canes (inner diameter: M- and L-sizes) were fixed to perforated veneer boards (30 cm × 15 cm) with a vinyl-coated wire. All bamboo canes were 20 cm long, with one end closed by the node and opened at another side. Traps were attached to tree trunks approximately 1.5 m above the ground and were separated for more than 20 m distance along the road or in the forest. Traps were installed from mid-July to mid-September to cover the whole nesting period of *I*. *harmandi* yearly. Sixty traps were used in 2010, 24 in 2011, 30 in 2012, 50 in 2013, and 20 in 2015.

We surveyed traps to collect the nest of *I*. *harmandi* every 2 or 3 d during the early and peak-nesting periods and every 5–7 d after the peak-nesting period. The presence of *I*. *harmandi* nests in the bamboo cane was checked by inserting a grass stem into the cane to probe the temporal plug placed inside or directly detected by its final closure plug projecting from the cane’s entrance. The nest only with temporal plug usually indicates that the female wasp still engages in the nesting activity; however, the exact stage of nesting was uncertain, and nesting was sometimes completed when nest desertion occurred at the time of building final closure plug. Thus, we principally collected all canes containing nests found in traps, except for nests where provision did not begin; then, they were brought back to the laboratory and kept in plastic bags stored in a cool box. See Table A in S1 File for numbers of *I*. *harmandi* nests collected during the study.

### Nest recording

Canes were split vertically in half on the day of collection, and the following aspects of the nest, including nesting phase, brood size, development stage of brood, mortality, and prey, were recorded.

#### Nesting phase

Nesting sequence was divided into the following five phases; (i) pre-hunting (temporary plug only), (ii) egg-laying (wasps consecutively laying several to a dozen eggs on each prey), (iii) provisioning (wasps storing some prey, rarely laying eggs on them), (iv) closing (compressing temporal plug and making final closure plug), and (v) completed (wasps already completing the nest). All sampled nests were assigned to one of these nesting phases (Table A in S1 File). However, some nests of phase (iii) contained egg-attached prey placed separately from several egg-laid prey in the deepest part of the cavity [23] (see Table B in S1 File). This means that the brood size of the nest of phase (ii) to which we assigned at the time of collection may be underestimated (see below).

#### Brood size

We counted the number of live or dead eggs, larvae, and prepupae (cocoons) found in the nest. The total number of wasp offspring was regarded as brood size at the nesting phase.

#### Developmental stage of broods

**Fig 1:**
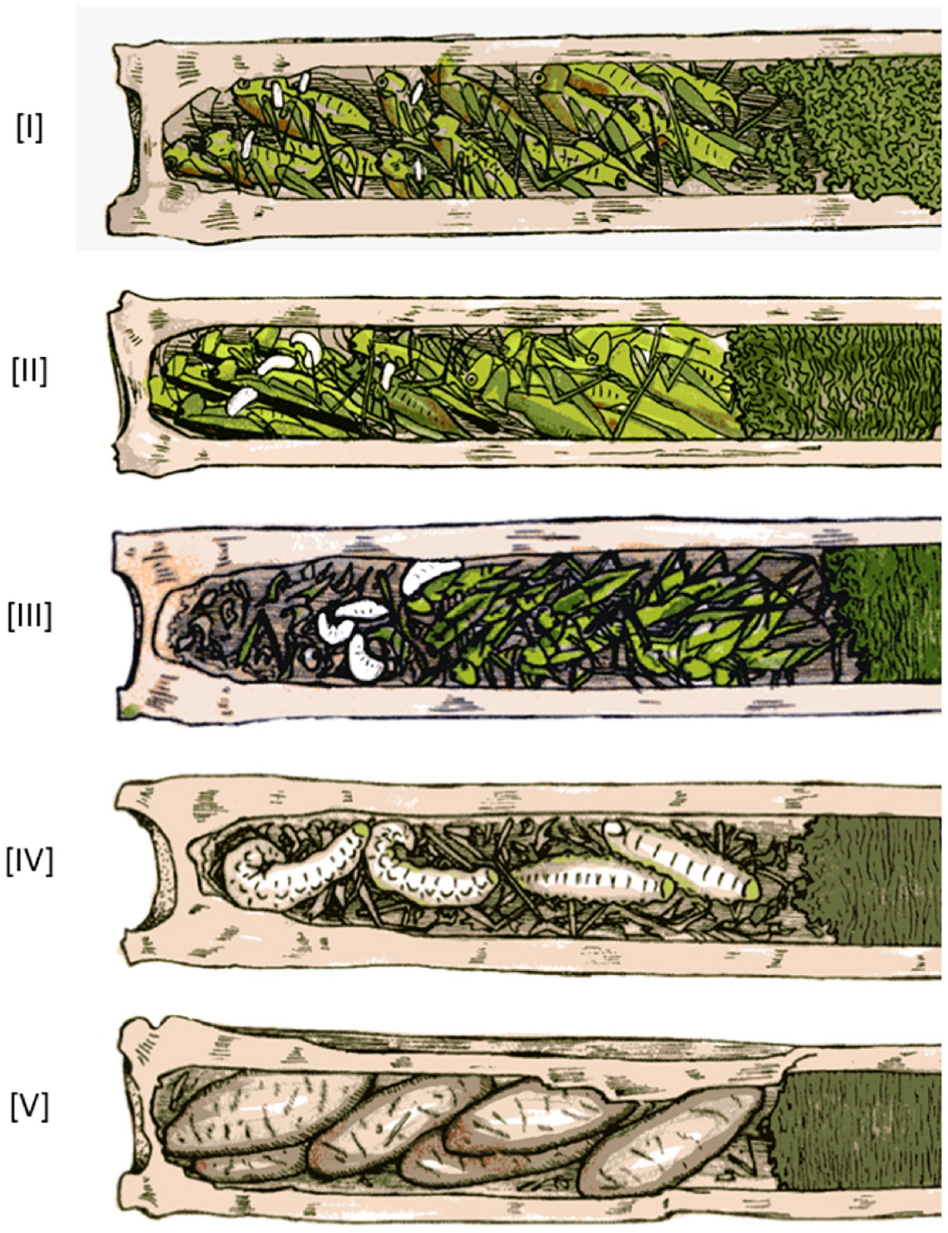
Illustrative presentation of different developmental stages of brood in *Isodontia harmandi* nest. Developmental stage [I], eggs, which are singly laid on each prey by female wasp; [II], hatchlings, which emerged and begin to consume their own prey; [III], middle-sized larvae, which move around and consume one and another prey in the brood cell; [IV], full-grown larvae have consumed almost all prey; and [V], almost all larvae spun their own cocoons.

Some nests of *I*. *harmandi* contained the wasp offspring of different developmental stages, such as eggs and larvae. Therefore, the developmental stages of the entire brood were classified into five categories according to developmental stages of individual offspring and its combination: [I] eggs, [II] eggs plus hatchlings or hatchlings (hatchling is a tiny larva and remained on the prey where its egg hatched), [III] most brood are middle-sized larvae (feeding in the crowded prey items away from the first prey), [IV] most broods are full-grown larvae (almost consuming up prey in the nest), [V] most brood prepupae (cocoons) (Fig 1). The developmental stage of the brood did not correspond to the nesting phase. For example, even though the developmental stage of brood was [I], the nesting phase was either (ii) or (iii) (see Table A in S1 File).

#### Mortality

Nests often suffered from attacks by various predators or parasitoid insects, ant predation (*Crematogaster teranishii* and *Monomorium intrudens*), and dipteran brood parasitism (*Amobia distorta* and Phoridae spp.). The larvae also died because the prey spoiled in the nest, got moldy, and was dried at sampling. Therefore, we inspected the nest contents in detail to exclude the data of nests associated with these mortality factors other than larval interaction.

#### Prey

Four orthopteran species were mainly used as prey by *I*. *harmandi* in the study sites; three meconematid species, *Cosmetura fenestrate*, *Leptoteratura albicornis*, and *Xiphidiopsis subpunctata*, and one tettigonid species, *Hexacentrus hareyamai*. Species, developmental stage (young or adult), sex, and larval consumption (consumed or intact) of each prey were recorded. If the prey is not consumed by the wasp larvae (i.e., intact), its fresh weight is measured using an electronic analytical scale (Shimadzu Corp., ATX-224), up to a minimum weighing unit of 0.1 mg. Total prey weight per brood is the sum of the weight of each prey item provided in nests where provisioning has been completed (i.e., nesting phases (iv) and (v)). However, the developmental stages of brood in these nesting phases varied from [II] to [V]. Because we could not measure prey weight for the nest of stages [IV] and [V] where almost all prey has been consumed, we used nest data of stages [II] and [III] where prey consumption by wasp larvae has progressed little. If some prey items were consumed and hence could not be weighed, we estimated their weight by using linear relationships between the number and weight of intact prey items. These data were obtained for sex/age groups of each prey species from all nests in which intact prey items were found (see S1 Fig; Table C in S1 File). Then, we calculated a total prey weight per brood by summing up the weight of intact prey and the estimated weight of consumed prey within a nest. Total prey weight per brood was determined for 69 nests (ten female nests, 40 male nests, and 19 sex-unknown nests; Table D in S1 File).

### Determination of clutch size

In *I*. *harmandi*, it is challenging to accurately determine the total clutch size laid by a female in a single nest because some eggs can be laid after the early consecutive oviposition, and we cannot assume any cannibalism among larvae after hatching. Clutch size can, therefore, be only determined for nests that satisfy two criteria: (a) provisioning phase (iii) had completed (i.e., phases (iv) and (v)) and (b) brood development is still at stage [I] or [II] (i.e., individuals of the brood are either eggs or hatchlings). We obtained 33 nest data satisfying both criteria (a) and (b) (five female broods, 19 male broods, and nine sex-unknown broods; hereafter referring to as strict data set). To increase available data on clutch size, we analyzed the relationship between the provision order of prey and the cumulative numbers of eggs laid, based on data of the nests with criteria (a) and (b). The curve of cumulative egg numbers is saturated with the increasing number of prey provisioned by wasps (S2 Fig; Table B in S1 File). Of 130 eggs laid in 19 nests, more than 99% (129 eggs) were found among the clump of prey when the 23rd item was carried into the cavity. Therefore, we can evaluate 99% of the total clutch size, even if we include a nest which has not been completed yet but already provisioned with more than 23 prey items. In this way, we obtained twelve additional data on total clutch size of *I*. *harmandi* (four data each for female, male, and sex-unknown brood; hereafter referring to as broad data set, combining these and strict data). See S2 File for which nests used as clutch size data.

### Rearing *I*. *harmandi* brood

#### Whole brood rearing

We reared the whole brood of nests to determine whether brood reduction occurs under natural prey conditions; unparasitized nests were used for rearing. Rearing for this purpose is conducted using nests that were sampled in 2010 and 2015. In total 39 broods were used for analysis (seven female broods, 21 male broods, and 11 sex-unknown broods). These were all in the range from developmental stages [I] to [III] and from nesting phase (iii) to (v).

The whole brood of the nests was reared in the original bamboo canes or other containers. After pulling out all wasp offspring and prey (with wasp eggs or hatchlings) from the nest cavity, we recorded and put them back into the original cane or moved them to the rearing container. Therefore, each experimental brood had the original prey amount provided by female wasps (see S3 File for detailed conditions of broods). Original bamboo and plastic cases with transparent cover (146-mm length × 26-mm width × 15-mm depth) were used mainly in 2010, and plastic Petri dishes (35-mm diameter × 8-mm depth) were used mainly in 2015. We raised them until all surviving larvae began spinning cocoons under an air-conditioned laboratory. Then, all cocoons were individually reared until adult emergence in the method described below. We counted the number of surviving offspring in each brood at the cocoon spinning stage and adult emergence.

#### Multi-sibling rearing

In 2012 and 2013, we conducted rearing experiments to clarify the cause of brood reduction, if it occurred. We reared the wasp larvae in a group of 3–9 siblings extracted from unparasitized nests under the time-lapse recording at an interval of 5 min (in 2012) or 3 min (in 2013) using a digital camera (RICHO, CX4). Groups of siblings were separately placed into plastic cases with transparent cover (146-mm length × 26-mm width × 15-mm depth) in 2012 or plastic Petri dishes (35-mm diameter × 8-mm depth) in 2013, provided with 0.40 ± 0.21 g (mean ± SD) per-capita prey. Group rearing and time-lapse recording were continued until all surviving larvae spun their cocoons. We checked whether cannibalism occurred among siblings by viewing time-lapse images taken for each group. Also, we checked whether there were or were not corpses of larvae left in the rearing case at the end of the experiment. Multi-sibling rearing was conducted in 19 groups (five groups of female siblings, ten male siblings, and four sex-unknown siblings).

#### Determination of brood sex

As most (97.3%) *I*. *harmandi* nests were single-sex brood (35.6% female brood, 61.6% male brood, and 2.7% mix-sex brood, n = 209; Fujita and Endo [Unpublished]), we can safely determine the brood sex based on the knowledge about the sex of an individual of the brood. To determine the brood sex, we raised all or part of the brood for nests containing viable larvae of all samples. All or part of the brood were moved to the rearing container (described above) separately in a brood unit and were raised until spinning under the same condition. Then, cocoons were individually transferred to glass vials (27-mm diameter × 55-mm height). These glass vials containing cocoons were kept in the outside storeroom for overwintering. The following year, we pinned adult wasps that emerged from cocoons and determined the sex. When we could not obtain any adult wasps of a brood, the brood was assigned to “sex-unknown.” A reason for no adult specimen of brood is that we released some nests containing cocoons into the study site to prevent population decline of *I*. *harmandi* after 2012.

### Statistics

Our data were obtained from different study sites conducted in different years, and we principally used a generalized linear mixed model (GLMM) to test any statistical significances, treating different year data as random effects. We are also interested in the sexual difference in the brood because differential relatedness between female and male brood arisen from hymenopteran sex determination mechanism may influence the parental investments and performance of siblings. However, we could not identify the sex for all broods; thus, we had the third sex-category, “sex-unknown brood.” Then, we conducted multiple comparisons for sexual differences in any variables using Tukey method.

We first conducted GLMM to examine the effect of brood sex categories on the initial clutch size and the number of cocoons in a nest. Response variables are initial clutch size and cocoon number, assuming a Poisson distribution with log link, using glmer function in the “lme4” package. Multiple comparisons between sex categories of brood were performed by glht function in “multcomp” package. Analyses on clutch size were conducted for both strict and broad data sets (see above). When analyzing brood reduction in *I*. *harmandi* nests, we examined whether brood’s developmental stage, nesting phase and sex categories have significant effects on brood size using GLMM. This is applied to all available data (n = 318); brood reduction was detected through multiple comparisons as the significant effect of different developmental stages on brood size. GLMM analysis for brood reduction was also performed to each brood-sex category, separately (n = 39 for female brood, n = 146 for male brood, and n = 133 for sex-unknown brood).

Next, we conducted GLMM to examine parental investment differences in different brood sex categories before testing the clutch size effect. We used the lmer function implemented in the “lme4” package with total prey weight as response variable, assuming a Gaussian distribution and an explanatory variable as sex-categories of brood. Nest data with estimated total prey weight were used in this analysis (n = 69). As female wasps deliver a massive amount of prey to their nest after they have laid several to a dozen eggs, we clarified whether the food amount of each nest estimated as total prey weight depends on clutch size in that nest. This was conducted using GLMM with a response variable as total prey weight and explanatory variables as clutch size and brood sex categories. Data were used for the nest with total prey weight and clutch size information (n = 33). Additionally, we also performed GLMM using prey amount per individual wasp offspring, i.e., per-capita prey weight instead of total prey weight as a response variable. These analyses were conducted for overall broods and different brood sex categories separately, except for female brood, using the lmer function. As yearly data for female broods were small, and it could not allow conducting GLMM, we analyzed the effect of clutch size in female brood on total and per-capita prey weight by the general linear model (GLM). GLMs were performed by glm function.

For whole brood-rearing experiments, multiple logistic regression analyses by GLMM were conducted for survivals of wasp offspring during two periods as response variables, separately; larval survival for the period from egg/larva to cocoon stage and cocoon survival for the period from cocoon to adult emergence. We set prey weight used in rearing each brood, number of eggs and larvae (brood size) and sex categories of brood as explanatory variables, and different years (2010 and 2015) as random effects, using the glmer function. In this analysis, identification of the nest was also treated as random factor, as survival of individual wasp offspring from same nest was included. Numbers of survived (= 1) and dead (= 0) offspring within a brood were assumed to be a binomial distribution.

We checked overdispersion for Poisson models and normality for Gaussian models by check_overdispersion and check_normality function in “performance” package, respectively. Collinearity among explanatory variables were also checked by check_collinearity function in the same package. We conducted all statistical analyses using R v.4.0.5 [32]. See S4 File for R script used in this study and S2 and S3 Files for csv files used in the R script.

## Results

In five years during 2010 to 2015, 495 nests of *I*. *harmandi* were obtained from study sites (Table A in S1 File). There were 88 nests (17.8% of all nests) containing no prey and wasp offspring and eight nests (1.6%) containing only prey, both closed by a temporal plug. The latter was supposed to be either nests where females failed to oviposit or nests attacked by some brood-parasitic insects, such as phorid flies that suck wasp eggs before maturation, but we failed to detect them. In 81 cases of nests (16.4%), we found some signs of predation and parasitism or deteriorated conditions. The remaining 318 nests (64.2%) contained both prey and wasp offspring at various developmental stages of brood in different nesting phases. These were all single-celled nests and used for analyzing brood reduction along developmental stages of brood in the nest.

### Brood reduction

The mean clutch size of different sex categories of nests in the pooled data were 9.2 ± 2.9 (SD) (range: 5–13, n = 5), 7.3 ± 2.2 (3–12, n = 19), and 6.8 ± 2.2 (2–9, n = 9) for female, male, and sex-unknown broods, respectively (S3 Fig). There were no differences in the clutch size between different sex categories of brood for strict data set (*p* = 0.34 for female *vs*. male broods, *p* = 0.26 for female *vs*. sex-unknown broods, and *p* = 0.89 for male *vs*. sex-unknown broods; Table A in S5 File). The results were almost the same for broad data set (Table A in S5 File). Mean number of cocoons in a nest were 4.2 ± 1.6 (2–8, n = 17), 4.3 ± 1.4 (1–7, n = 39), and 3.9 ± 1.6 (1–7, n = 34) for female, male, and sex-unknown broods, respectively (S4 Fig). There were no significant differences in cocoon numbers between different sex categories of broods (*p* > 0.7 for all pairwise comparisons; Table B in S5 File). Thus, in average 54% ((9.2–4.2)/9.2), 41% ((7.3–4.3)/7.3), and 43% ((6.8–3.9)/6.8) of initial clutch were lost during developmental stages from egg or larvae to cocoon, for female, male, and sex-unknown broods, respectively.

**Fig 2:**
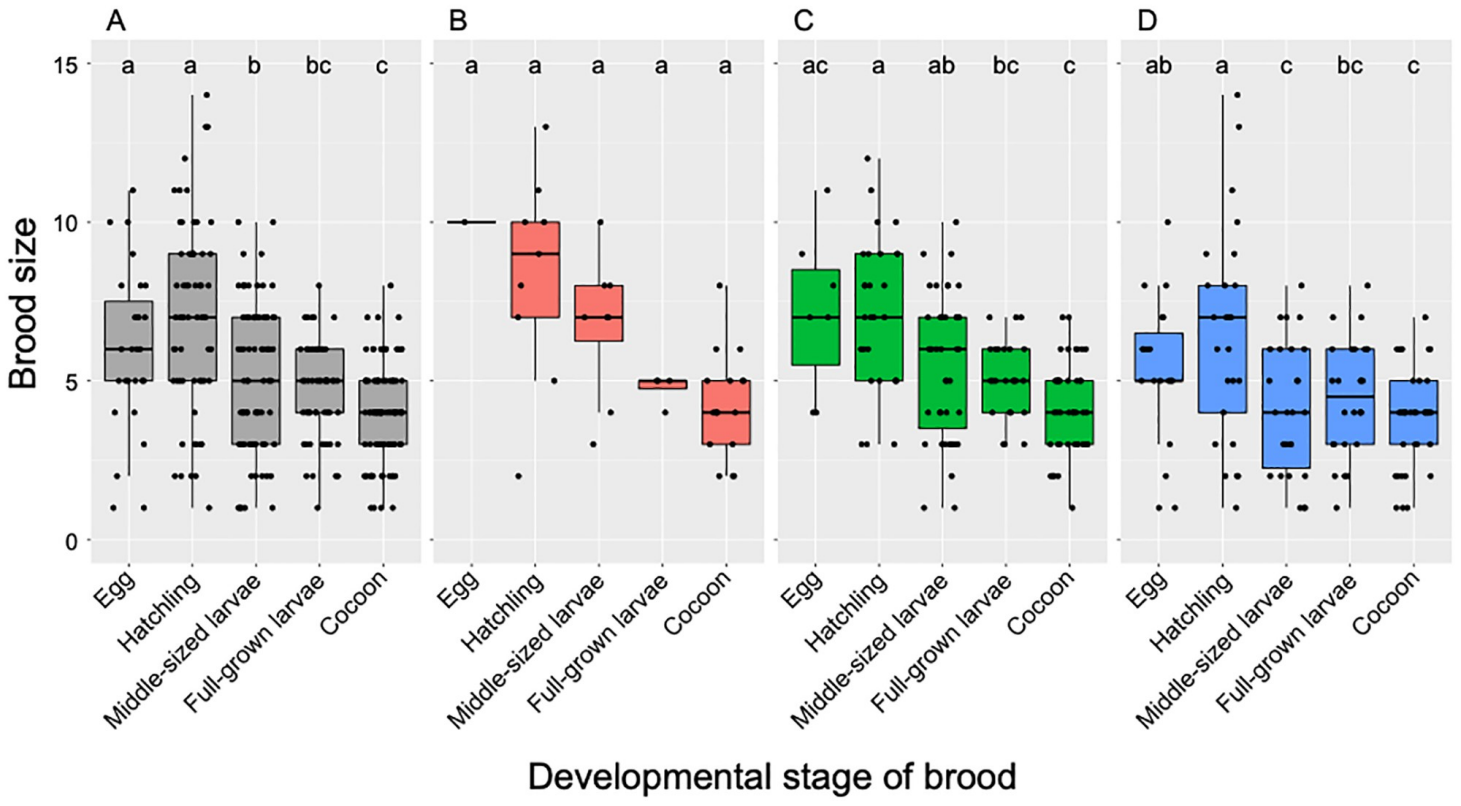
Changes in brood size along the developmental stages of brood in *Isodontia harmandi*. Brood size data for five years during 2010 to 2015 were pooled for different sex categories of broods. Different letters indicate statistical significance in brood size between pairwise comparison of developmental stages of brood.

The observed number of wasp offspring in a nest decreased as developmental stages of brood in all sex categories of nests progressed (Fig 2; Table E in S1 File). As a whole, significant brood reductions occurred in early and later stages of larval development (*p* < 0.001 for hatchling vs. middle-sized larvae stage and *p* = 0.004 for middle-sized larvae vs. cocoon stage; Table C in S5 File). There were differences in brood size between sex-unknown brood and female or male brood (*p* = 0.005 for female vs. sex-unknown brood and *p* = 0.02 for male vs. sex-unknown brood). When analyzing separately for brood-sex categories, significant difference among developmental stages was not detected in female broods. Brood size gradually decreased in male broods (*p* = 0.05 for hatchling vs. full-grown larva stage and *p* = 0.03 for middle-sized larva and cocoon stage). Significant brood reduction was detected during hatchling and middle-sized larva stage in sex-unknown broods (*p* < 0.001). Nesting phase had no significant effect on brood size for overall brood and all brood-sec categories (Table C in S5 File).

### Relationship between clutch size and total prey weight

Total prey weight varied from 0.81 to 5.50 g among nests with an average of 3.09 g. Female brood had on average 3.91g prey, male brood had 2.97 g, and sex-unknown brood had 2.90 g. Thus, female broods were more heavily provisioned than male or sex-unknown broods (*p* = 0.06 marginally significant for female vs. male brood and *p* = 0.04 for female vs. sex-unknown brood; S5 Fig; Table D in S5 File).

**Fig 3:**
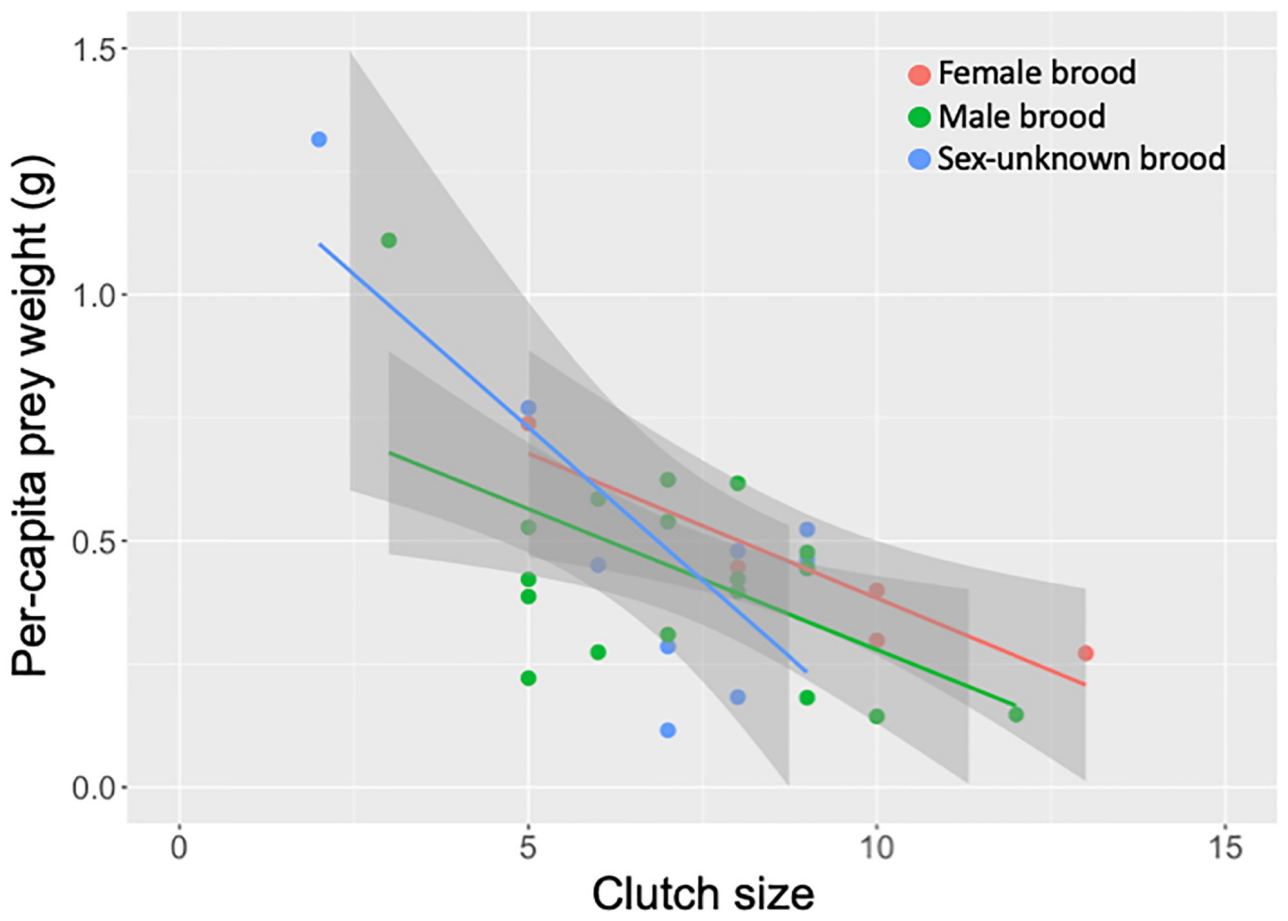
Relationship between clutch size and per capita prey weight in different sex categories of brood in *Isodontia harmandi*. Per-capita prey weight significantly decreased with clutch size for each sex category of brood. Lines and shaded parts indicate linear regression and 95% confidence interval for each sex category of brood.

Both total prey weight and clutch size of nests were determined for 33 of 69 cases. Surprisingly, there was no relationship between clutch size and total prey weight for any sex categories of brood in *I*. *harmandi* (S6 Fig; *p* > 0.05 for all pairwise comparisons; Table E in S5 File). As a result, per-capita prey weight significantly decreased with clutch size for each sex category of brood (Fig 3; *p* < 0.001 for overall, *p* = 0.02 for all sex categories; Table F in S5 File). There were no significant differences in both total prey weight–clutch size relationship and per-capita prey weight–clutch size relationship among brood-sex categories (*p* > 0.3 for all pairwise comparisons; Tables E and F in S5 File).

### Relationship between wasp offspring survival and total prey weight

In whole brood-rearing experiment, 156 larvae (58.9%) of 265 wasp offspring from 39 nests survived until cocoon stage and 101 cocoons (64.7%) of them successfully emerged into adult stage. Brood reduction occurred in 30 nests (77% of nests) during larval stage and in 23 nests (59%) during cocoon to adult emergence. All brood members were lost in 8 nests (21%) (see S3 File).

**Fig 4:**
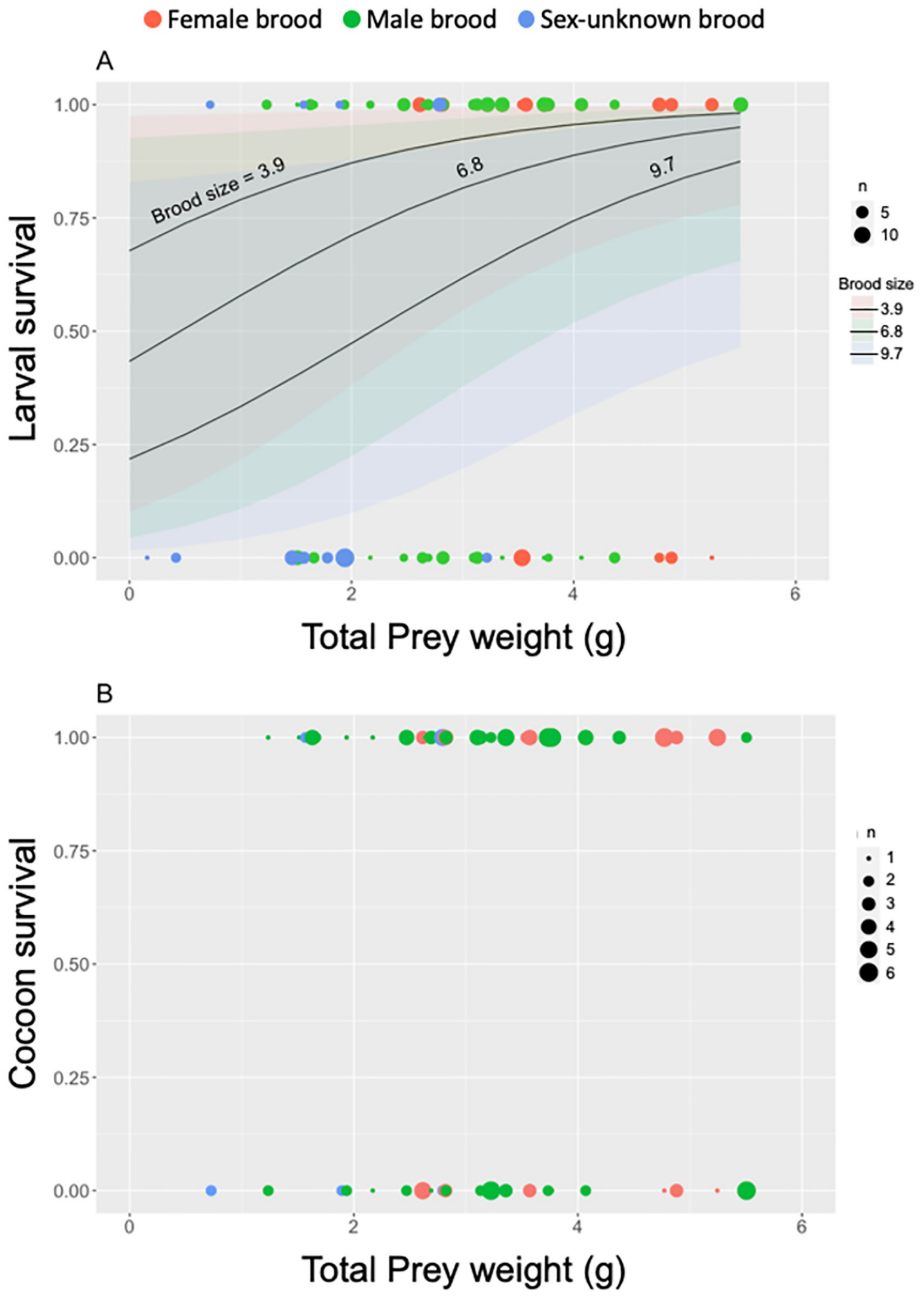
Relationship between survival of individual wasp offspring and total prey weight per brood in whole brood rearing experiment. Survival of wasp offspring were shown during eggs or early larvae to cocoon stage (A) and during cocoon to adult emerging stage (B). Logistic regression lines with 95% confidential intervals were fitted to female brood data at three values of brood sizes (3.9, 6.8, and 9.7) for larval survival, indicating a significant positive effect of total prey weight and a significant negative effect of brood size on larval survival. There was no relationship between cocoon survival and total prey weight or brood size. There was no difference in larval survival between female and male brood. But larval survival of sex-unknown broods differed significantly from male broods and marginally from female broods (Table G in S5 File). Dot size indicates the number of wasp offspring.

Results of logistic regression analysis for larval and cocoon survival in this rearing experiment are shown in Fig 4. We confirmed a significant positive effect of total prey weight and a significant negative effect of brood size on the survival of wasp egg/larva (Fig 4A; *p* = 0.05 for total prey weight and *p* = 0.005 for brood size; Table G in S5 File). However, total prey weight and brood size had no significant effect on the survival during cocoon to emerging adult stage (Fig 4B; *p* > 0.6 for both variables; Table H in S5 File). There was no difference in the larval survival between female and male brood, but a significant difference between sex-unknown and male brood (*p* = 0.82 for female vs. male brood and *p* = 0.03 for sex-unknown vs. male brood; Table G in S5 File). There were no differences in the cocoon survival among brood-sex categories (*p* > 0.6 for all pairwise comparisons; Table H in S5 File).

### Occurrence of sibling cannibalism

In rearing experiments using multiple offspring of wasps, cannibalism was observed by watching the time-lapse recording of a digital camera. Sibling cannibalism occurred in 14 (74%) of 19 groups containing multiple larvae or eggs (Table 1). More than half of the wasp offspring died in this experiment (45/84 = 54%). Most of these deaths (34/45 = 76%) were caused by sibling cannibalism (Table 1). We could not determine all mortality factors for individual larvae because ten larvae were missed by time-lapse recording (3 or 5 min interval). Bodies of these larvae were not found in the rearing cases when the contents of the cases were carefully checked at the end of the experiments. Another larva remained as a corpse.

**Table 1:** Outcomes of individual broods in partial brood rearing experiments performed under frame-by-frame recording by digital camera.

Year	Exp. No.	Used nest ID	Initial number of wasp offspring	Final number of wasps which				
				survived to cocoon stage	died of cannibalism	died from unknown cause	disappeared	Sex of brood
2012	1	388	3	1	2	0	0	Unknown
	2	388	3	1	2	0	0	Unknown
	3	389	8	1	5	0	2	Male
	4	393	3	3	0	0	0	Male
	5	396	3	2	1	0	0	Male
	6	394	8	2	3	0	3	Male
	7	405	4	4	0	0	0	Female
	8	404	8	3	5	0	0	Female
	9	400	3	1	1	0	1	Female
	10	400	3	2	0	0	1	Female
	11	410	3	2	1	0	0	Male
	12	410	3	1	2	0	0	Male
	13	424	3	3	0	0	0	Male
	14	424	3	2	1	0	0	Male
	15	432	3	3	0	0	0	Unknown
	16	429	3	1	1	0	1	Female
2013	17	466	8	3	4	0	1	Unknown
	18	467	8	1	5	1	1	Male
	19	503	4	3	1	0	0	Male
Total (%)			84	39 (46.4)	34 (40.5)	1 (1.2)	10 (11.9)	

Cannibals were usually larger than the victims. Victims were often hatchling remaining on prey to which they were attached as egg or even an egg (S7 Fig): however, cannibalism also occurred between middle-sized larvae (S8 Fig). After the cannibals touched the victim’s body with their mouthparts, they ate the victims without overt aggressive interactions (S7 Fig). In one case, a victim and its prey item that it was feeding were shared by two or three larvae, and cannibalistic consumption was initiated by one of them (S7 Fig).

## Discussion

This study demonstrated that there was a significant decrease in brood size of *I*. *harmandi* between the nests collected at the egg or hatchling stages and those at later stages even in healthy nests (Fig 2). In the nests of the later stage without obvious mortalities, we could rarely discover any corpse of the larvae likely corresponding to the brood loss from the early phase. In many other invertebrate studies, individual disappearance has been considered indirect evidence of cannibalism for dragonfly larvae [33] and wolf spiders [34]. We also confirmed that brood reduction occurred in the whole brood-rearing experiment (Fig 4). Furthermore, in multi-larvae rearing experiments, we observed many cases of sibling cannibalism. If we could regard ten cases of larval disappearance in this experiment as missed cases of cannibalism, percentages of wasps died of sibling cannibalism in the total population, and total mortality might jump up to 52% and 98%, respectively (Table 1). Therefore, we conclude that brood reduction routinely occurs by sibling cannibalism in the communal brood cell of *I*. *harmandi*.

A causal factor of sibling cannibalism in this species may be the shortage of prey provided by female wasps because per-capita prey weight became smaller in the nest with larger brood size (Fig 3), and fewer offspring survived until cocoon spinning under a smaller amount of prey and larger size of brood (Fig 4A). This strongly indicates that cannibalism may be controlled by food shortage or some mechanisms involving the response of the wasp larvae to this food condition, as suggested by early studies of this species [25–27]. Therefore, further studies that experimentally control both prey amount and brood size are needed to determine whether food shortage drives sibling cannibalism in *I*. *harmandi*, and to clarify how wasp larvae switched from prey consumption to cannibalistic behavior depending on available prey amount.

Cannibalistic behavior was often documented among the larvae of gregarious parasitoid wasps sharing a single host when superparasitism occurred [35–37]. In solitary wasps, intraspecific brood parasitism often occurred [16, 38]. Therefore, if one female wasp would lay egg(s), invading the nest where another female had already prepared eggs and prey, the same situation as superparasitism in parasitoid wasps might arise in solitary wasps. Additionally, if intraspecific brood parasitism occurs in *I*. *harmandi*, we cannot deny the possibility that cannibalism occurs among wasp larvae from different broods. If so, a few eggs sporadically laid after consecutive egg-laying phases may be a suspected candidate for those of intraspecific brood parasites (Table B in S1 File). We believe, however, that this is unlikely for the following reasons; first, there was no previous observation of intraspecific brood parasitism in this species [22–27]. Second, we could not detect any traces of brood parasitism, such as breakage of temporal or final plug in our numerous samples of *I*. *harmandi* nests. Finally, even if brood parasite could enter and lay some eggs in the host nest, the parasite’s offspring would likely be consumed by host larvae growing larger unless the parasitic female wasp had eaten or damaged all host eggs or larvae.

Brood reduction by sibling cannibalism in *I*. *harmandi* is likely to solve the problem arising from parental overproduction [2]. In solitary wasps making “private” cell(s) in a nest, wasps usually lay an egg and store prey to provide for it in each cell and make a cell individually. Therefore, the problem of overproduction may be less important in these solitary wasps, even if female wasps first lay an egg before prey provisioning, as in the case of eumenid species, or lay an egg on the first prey as in the case of many sphecid wasps [28, 39]. They may not overproduce more than one offspring when prey resource becomes unavailable for them. However, the overproduction problem may probably become more important in a communal brood cell of *I*. *harmandi*. It was surprising in this context that we could not find any relationship between clutch size and the amount of provision (S6 Fig). In *Isodontia mexicana*, a congeneric species with sexual size dimorphism, female wasps provide greater numbers of prey to cells in which female-destined eggs were laid [40]. Though we found that female broods were also likely to be provisioned with a larger mass of prey than male broods in *I*. *harmandi* (S5 Fig), which adult female size was considerably variable but larger than male [22], total provisions were unrelated to the clutch size for both sex broods. This indicates that provisioning wasps may be confronted with the unpredictability of prey resource availability. Then, overproduction in this species may be explained by the resource-tracking hypothesis [1, 2]. Though we have no information about prey availability of this species in the field, wasps usually hunted large numbers of small meconematid preys, such as *C*. *fenestrate*, *L*. *albicornis*, and *X*. *subpunctata*, as Iwata [28] characterized this species as a small-prey user (average body weight: 0.090 g, 0.082 g, and 0.047 g, respectively; S1 Fig). However, wasps occasionally provisioned tettigonid prey, *Hexacentrus hareyamai*, 1.5–2.8 times heavier than these small prey species (0.133 g; S1 Fig). Prey use of such a wide size range may accelerate the unpredictability of prey availability by including large prey items, such as a bonus.

Hatching asynchrony has been for many decades focused as a mechanism facilitating brood reduction by sibling rivalry [4, 41, 42]. Several studies showed that hatching asynchrony also played an important role in reducing sibling competition [10, 43, 44]. However, it is uncertain whether sporadic oviposition in the later stage of provisioning in *I*. *harmandi* plays a role presumed by asynchronous hatching in these insects. Rather, successive egg-laying in this species is possibly a trait derived from the mode of oviposition at temporally spaced intervals in the species where provisioning follows egg-laying in every brood cell [45]. It is fascinating to understand why this distinct behavior of successive egg-laying has evolved in this communal brood-cell making species. Making communal brood cells might offer selective advantages of effectively using the cavity space, which may be a limiting resource and quickly produce more offspring by omitting time-consuming tasks, such as constructing bulky partitions between brood cells. If so, successive egg-laying may be beneficial for female wasps to have more chance of reproducing rapidly in their short flight season. We did not know exactly how long it takes for females to lay eggs successively, but it would only take a day or a few days at the peak period, as we could find a newly completed nest in two or three days after the previous visit. Although it is unsure whether there is any mechanism to facilitate hatching asynchrony or individual difference in growth, size advantage seems to be not always necessary for cannibals to subdue the victims in this species. Some victims were small hatchlings or even egg stage of siblings (S7 Fig), but sibling cannibalism also occurred among the similar-sized larvae (S8 Fig). This was reflected by continuously reducing brood size during the later stage of development (Fig 2).

In his blackbird study, Forbes *et al*. [46] introduced measurement of “brood reduction efficiency,” which is composed of the mean per-capita input in victims of brood reduction. This is a measure for wasted food consumption, which eventually failed to be converted into a reproductively mature adult body. It is a significant fitness component of parents in birds, as cannibalism rarely occurs [1]. However, brood reduction is highly “efficient” in *I*. *harmandi*. Because brood reduction occurs routinely in the form of sibling cannibalism, prey consumed by the victims of brood reduction will eventually be assimilated by the cannibals except for respiration loss. Indeed, taking the results that many broods produced one or more offspring in whole brood-rearing experiments (see S3 File) into consideration, brood reduction by sibling cannibalism may facilitate avoiding the worst consequence of overproduction. Furthermore, when orthopteran prey compactly packed in brood cells often worsens, some rapidly growing larvae may be literally an “ice box” for other larvae [47].

Because kinship can promote the lowering threshold that victims give up their own reproduction, according to Hamilton’s rule, cooperative sibling cannibalism would likely occur among highly related animals [48]. Throughout this, we did not observe aggressive interactions during sibling cannibalism. This observation might imply that sibling cannibalism in *I*. *harmandi* attains cooperative aspects. However, we could not discern the sex differences in clutch size and brood reduction in this study, except for total prey weight. Thus, we should further study the roles of kinship in communal brood cells and evolutionary and ecological aspects of single-sex brood in *I*. *harmandi*.

## Supporting information

S1 Fig Relationships between the number and the weight of unconsumed prey items of four prey species. Lines and shadows show linear regressions of prey number on prey weight and the 95% confidence intervals, respectively for female prey (red), male prey (green), and immature prey (blue).
(TIF)

S2 Fig Egg-laying pattern in relation to provisioning order of prey observed in *Isodontia harmandi* nests. Relationship between frequencies of nests that female wasps laid and did not lay an egg on *i*-th prey item and prey provisioning order in 19 nests (A). Relationship between proportion of egg-laid prey items in the total of *i*-th prey items and prey provisioning order (B). Relationship between cumulative number of eggs laid among 19 nests and prey provisioning order (C). Female wasps laid an egg on the first prey in most of nests (94.7%). Egg-laying probability rapidly decreased with provisioning order until around 15th prey item, but sporadically continued on more than 20th prey item. Cumulative number of eggs reached 99% at 23rd prey.
(TIF)

S3 Fig Frequency distributions of clutch size in *Isodontia harmandi* nests collected for five years during 2010 to 2015. Data of different years are pooled for overall (A), female (B), male (C), and sex-unknown broods (D). Filled bars of each sex-category of brood indicate data of the nest where provisioning phase had completed (strict data set). Not-filled bars indicate data of the nest where provisioning phase had not yet completed, but provisioned with more than 23 prey. Sum of filled and not-filled bars are broad data set.
(TIF)

S4 Fig Frequency distributions of the number of cocoons in *Isodontia harmandi* nests collected for five years during 2010 to 2015. Data of different years are pooled for overall (A), female (B), male (C), and sex-unknown broods (D).
(TIF)

S5 Fig Frequency distributions of total prey weight per brood (g) in *Isodontia harmandi* nests collected for five years during 2010 to 2015. Data of different years are pooled for overall (A), female (B), male (C), and sex-unknown brood (D).
(TIF)

S6 Fig Relationship between clutch size and total prey weight in different sex categories of brood in *Isodontia harmandi*. There was no relationship between clutch size and total prey weight for any sex categories of brood.
(TIF)

S7 Fig Some examples of observations on cannibalistic behavior of larvae in *Isodontia harmandi*. Experiment 2013–1 (see Table 1). Initial number of wasp offspring was eight (six eggs and two hatchlings). Experiment started at 17:27 p.m. on July 27, 2013. Around 35 h and 20 mins after the experiment began, the largest larva [a], which had consumed about half of its own first prey, alighted from the prey and began to move around. Larva [a] touched other wasp larvae and some prey items around itself. About 35 mins later, larva [a] returned to the prey item, which it originally consumed, and soon approached directly the prey item with small wasp hatchling [f] (black arrow in A). Larva [a] began to consume hatchling [f] rather than the prey item. This event occurred without any overt aggressive interaction. Consuming hatchling [f] in 30 mins, larva [a] did not try to eat the orthopteran prey to which hatchling [f] had attached (see black arrow in B). Around 90 mins after this cannibalistic event, larva [b] (the 2nd largest) started moving around in the Petri dish. Simultaneously, larva [a] and [c] (the 3rd largest) also initiated moving. They got together the prey, which hatchling [e] was feeding. Larva [c] first consumed the prey item and then began to cannibalize the hatchling [e]. Larva [a] joined to cannibalize the hatchling [e] together (red arrow in B). Probably larva [b] also shared the victim. It took 33 mins to complete the consumption. After this communal cannibalism, three larvae, [a], [b], and [c], devoured the prey that hatchling [e] was eating.
(TIF)

S8 Fig Sibling cannibalism in the brood cell of *Isodontia harmandi*. A larva bending its body like mirror writing of a letter “J” was biting another larva at the thoracic part. Body lengths of both cannibal and its victim were around 20 mm. Scale bar indicates 10 mm at the center of the photo. Photo was taken just after opening the nest built in the bamboo cane trap by YI. Nest was sampled at August 8, 2010. Nest no. 65.
(TIF)

S1 File Nest and prey data of *Isodontia harmandi*. (XLSX)

S2 File Brooddata.csv. CSV file of all nest collections. This csv file provides data for R script.
(CSV)

S3 File Wholebroodrearingdata.csv. CSV file of whole brood-rearing experiment data. This csv file provides data for R script.
(CSV)

S4 File R script for analysis. (R)

S5 File Results of statistical analyses. (DOCX)

S6 File (PDF)
