# Supplementary figures and images for: Brood reduction caused by sibling cannibalism in Isodontia harmandi (Hymenoptera: Sphecidae), a solitary wasp species building communal brood cells
Source: PLoS One. 2022 May 18;17(5):e0267958. doi: 10.1371/journal.pone.0267958 (PMC9116661; doi:10.1371/journal.pone.0267958)

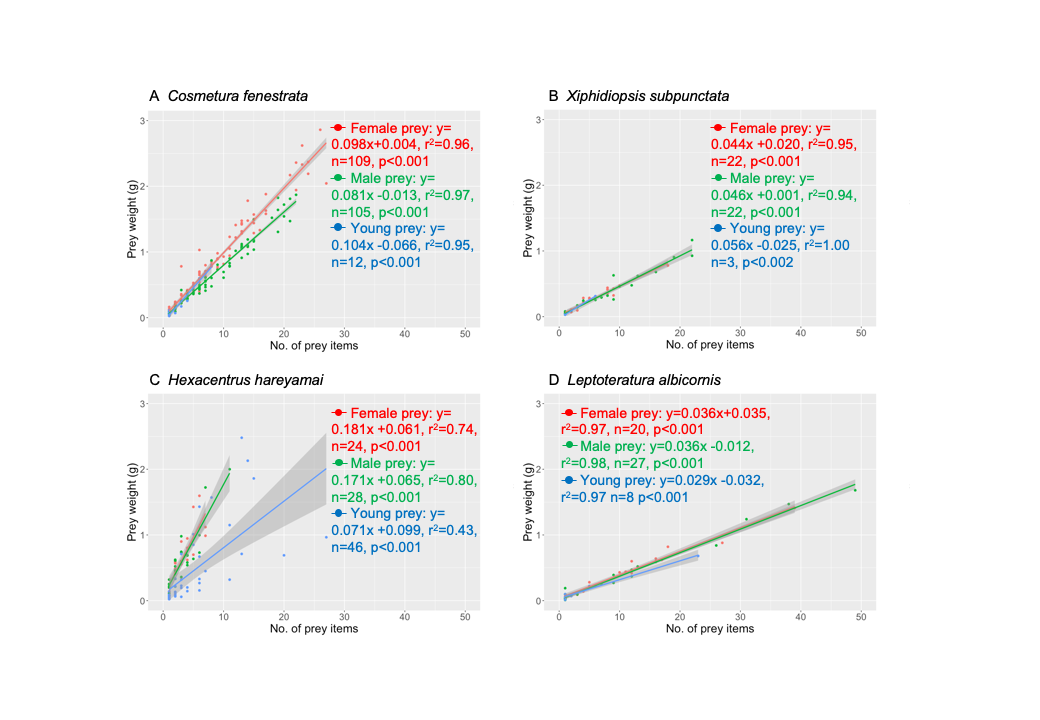

Supplement: S1 Fig — Lines and shadows show linear regressions of prey number on prey weight and the 95% confidence intervals, respectively for female prey (red), male prey (green), and immature prey (blue). (TIF) [file pone.0267958.s001.tif]

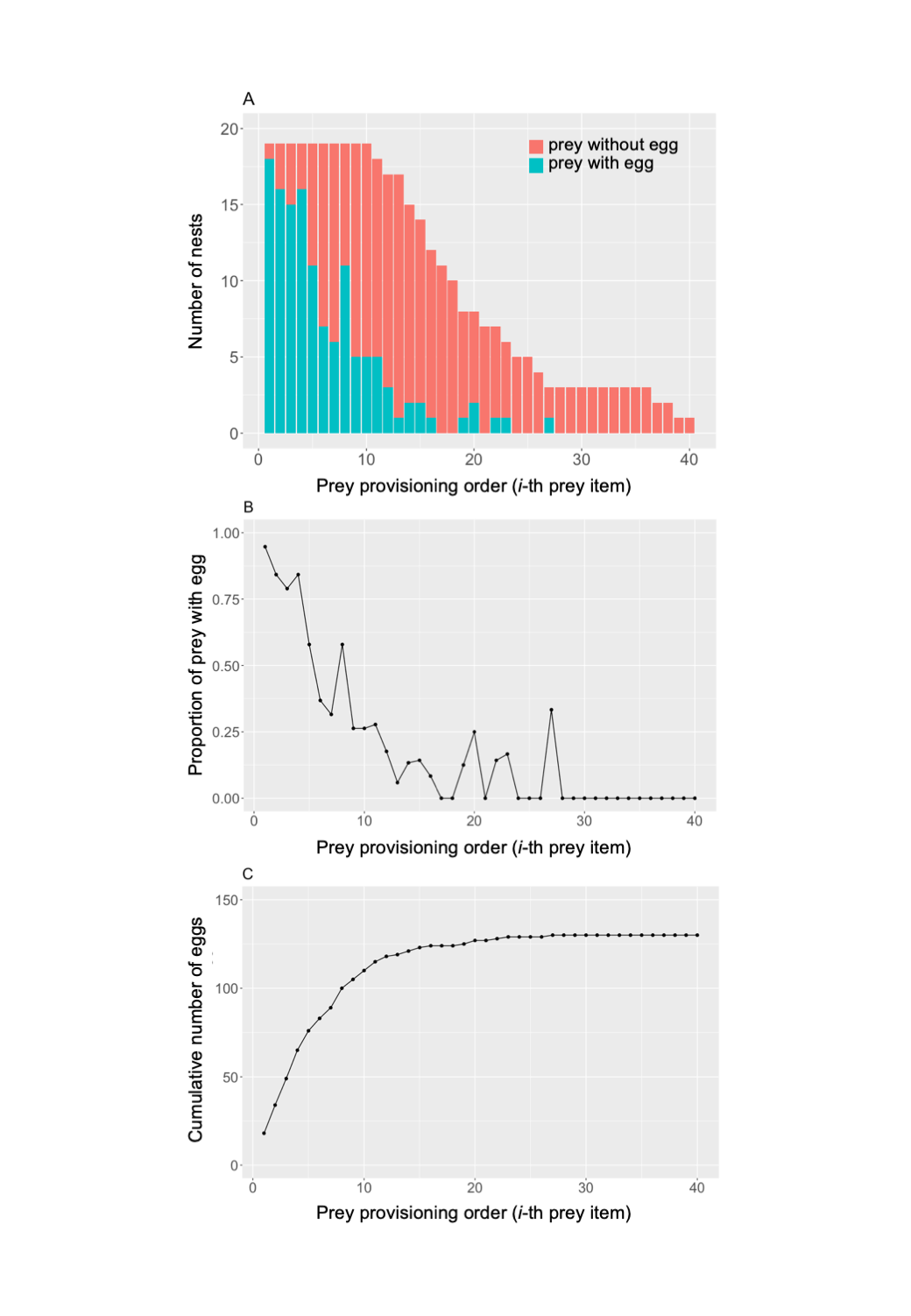

Supplement: S2 Fig — Relationship between frequencies of nests that female wasps laid and did not lay an egg on i-th prey item and prey provisioning order in 19 nests (A). Relationship between proportion of egg-laid prey items in the total of i-th prey items and prey provisioning order (B). Relationship between cumulative number of eggs laid among 19 nests and prey provisioning order (C). Female wasps laid an egg on the first prey in most of nests (94.7%). Egg-laying probability rapidly decreased with provisioning order until around 15th prey item, but sporadically continued on more than 20th prey item. Cumulative number of eggs reached 99% at 23rd prey. (TIF) [file pone.0267958.s002.tif]

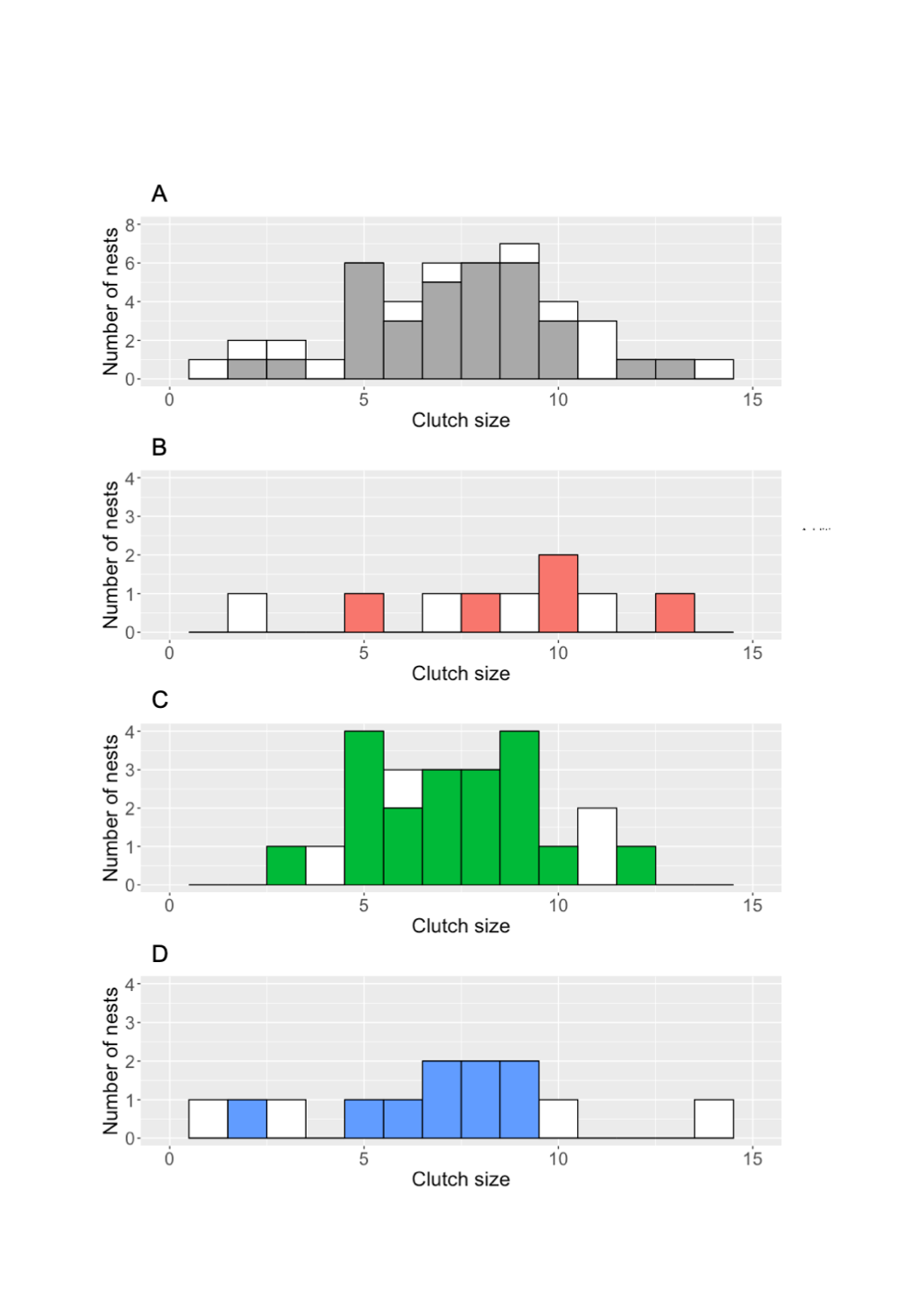

Supplement: S3 Fig — Data of different years are pooled for overall (A), female (B), male (C), and sex-unknown broods (D). Filled bars of each sex-category of brood indicate data of the nest where provisioning phase had completed (strict data set). Not-filled bars indicate data of the nest where provisioning phase had not yet completed, but provisioned with more than 23 prey. Sum of filled and not-filled bars are broad data set. (TIF) [file pone.0267958.s003.tif]

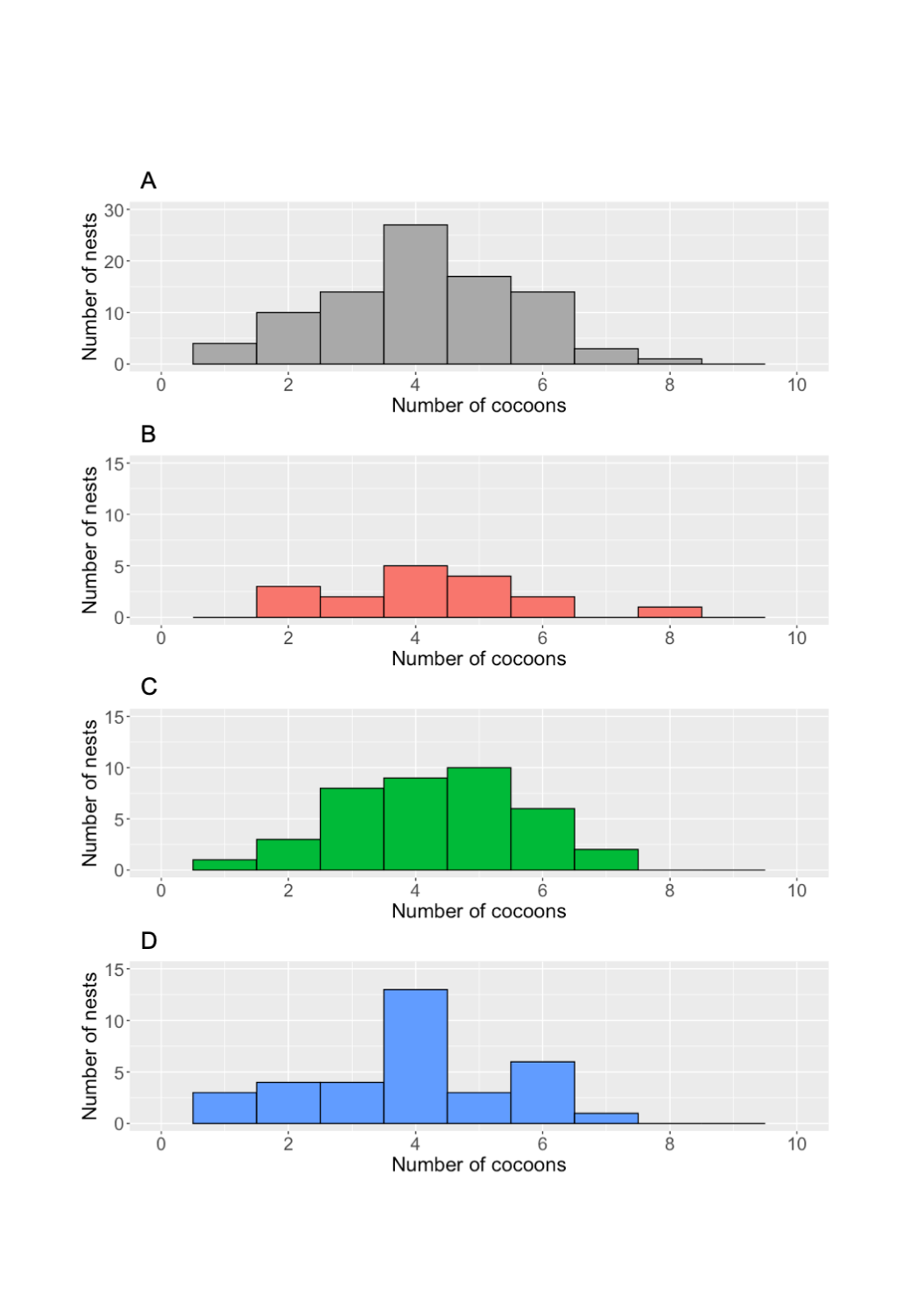

Supplement: S4 Fig — Data of different years are pooled for overall (A), female (B), male (C), and sex-unknown broods (D). (TIF) [file pone.0267958.s004.tif]

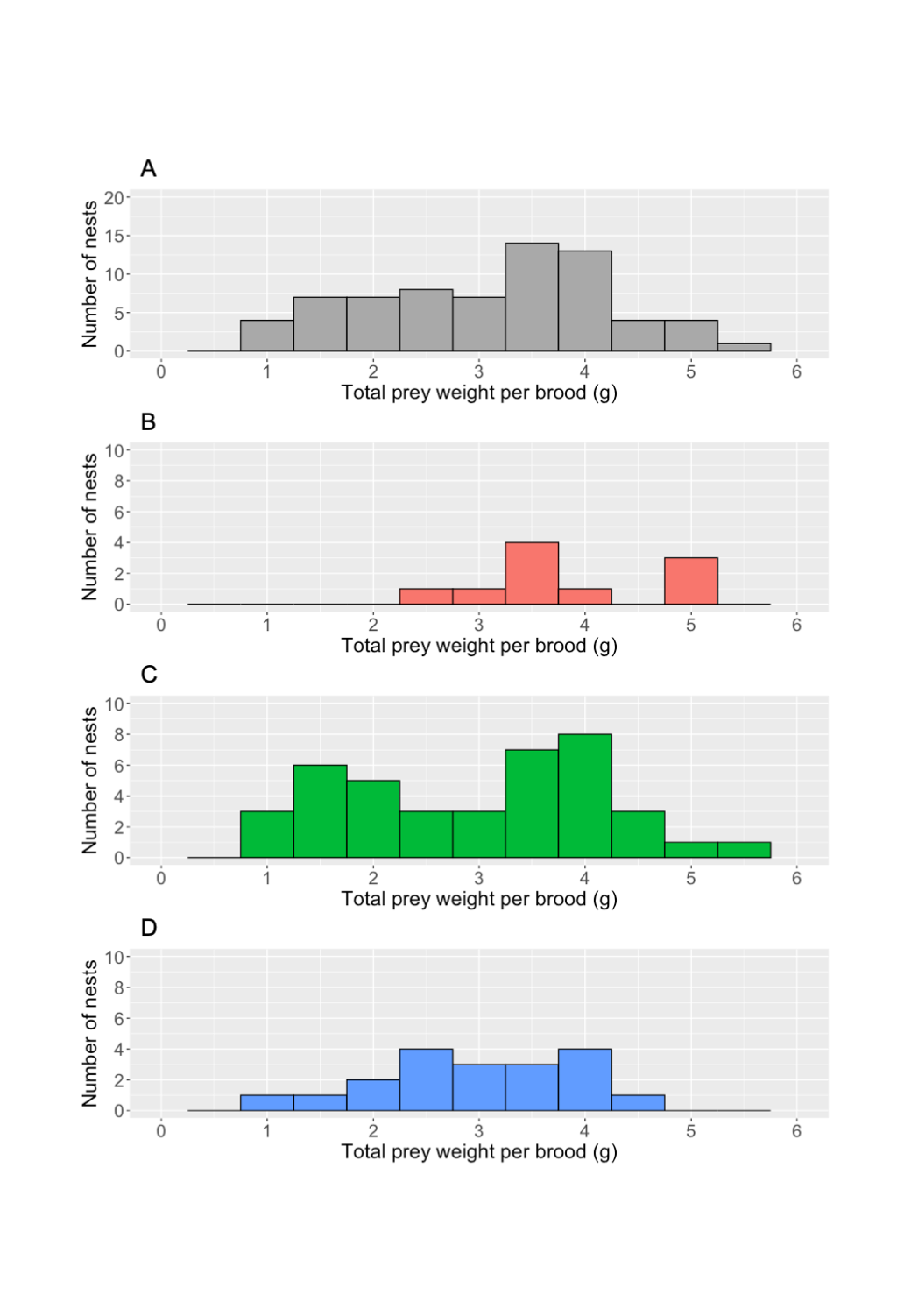

Supplement: S5 Fig — Data of different years are pooled for overall (A), female (B), male (C), and sex-unknown brood (D). (TIF) [file pone.0267958.s005.tif]

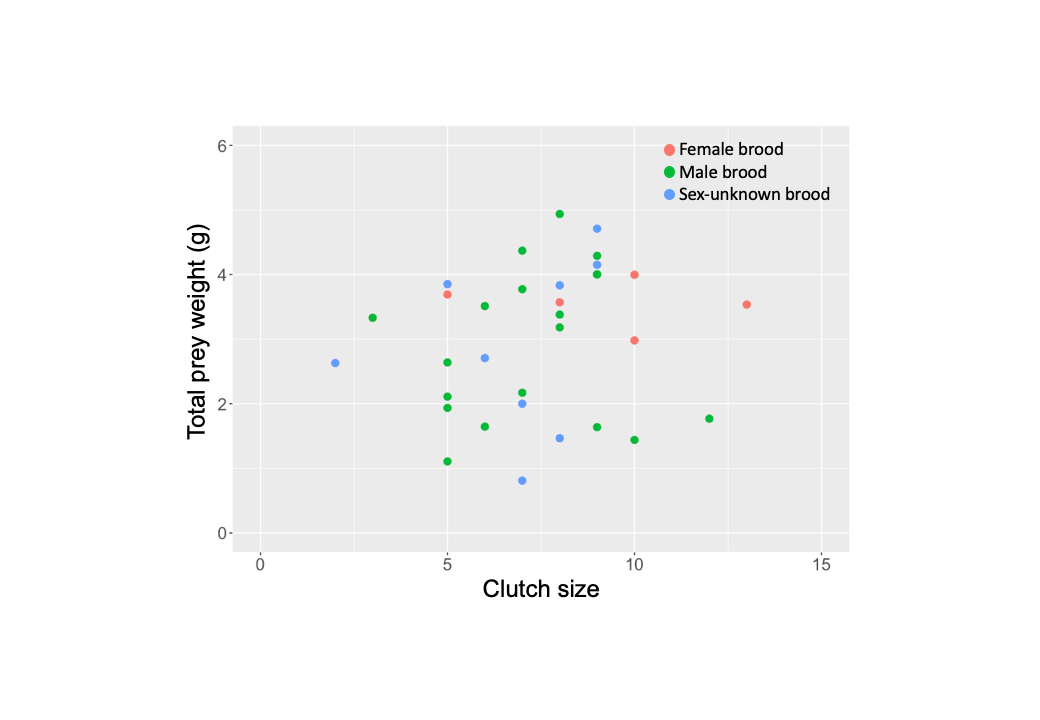

Supplement: S6 Fig — There was no relationship between clutch size and total prey weight for any sex categories of brood. (TIF) [file pone.0267958.s006.tif]

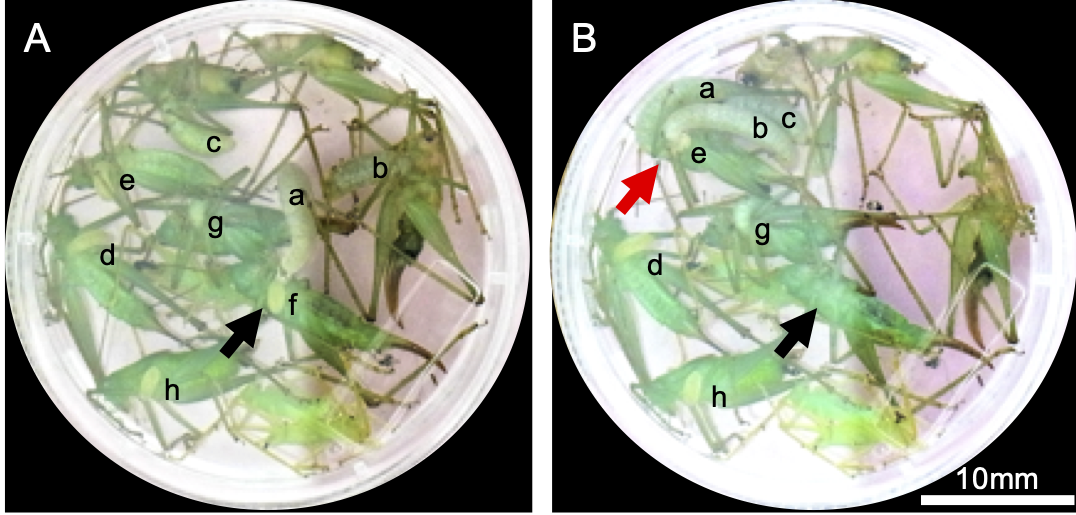

Supplement: S7 Fig — Experiment 2013–1 (see Table 1). Initial number of wasp offspring was eight (six eggs and two hatchlings). Experiment started at 17:27 p.m. on July 27, 2013. Around 35 h and 20 mins after the experiment began, the largest larva [a], which had consumed about half of its own first prey, alighted from the prey and began to move around. Larva [a] touched other wasp larvae and some prey items around itself. About 35 mins later, larva [a] returned to the prey item, which it originally consumed, and soon approached directly the prey item with small wasp hatchling [f] (black arrow in A). Larva [a] began to consume hatchling [f] rather than the prey item. This event occurred without any overt aggressive interaction. Consuming hatchling [f] in 30 mins, larva [a] did not try to eat the orthopteran prey to which hatchling [f] had attached (see black arrow in B). Around 90 mins after this cannibalistic event, larva [b] (the 2nd largest) started moving around in the Petri dish. Simultaneously, larva [a] and [c] (the 3rd largest) also initiated moving. They got together the prey, which hatchling [e] was feeding. Larva [c] first consumed the prey item and then began to cannibalize the hatchling [e]. Larva [a] joined to cannibalize the hatchling [e] together (red arrow in B). Probably larva [b] also shared the victim. It took 33 mins to complete the consumption. After this communal cannibalism, three larvae, [a], [b], and [c], devoured the prey that hatchling [e] was eating. (TIF) [file pone.0267958.s007.tif]

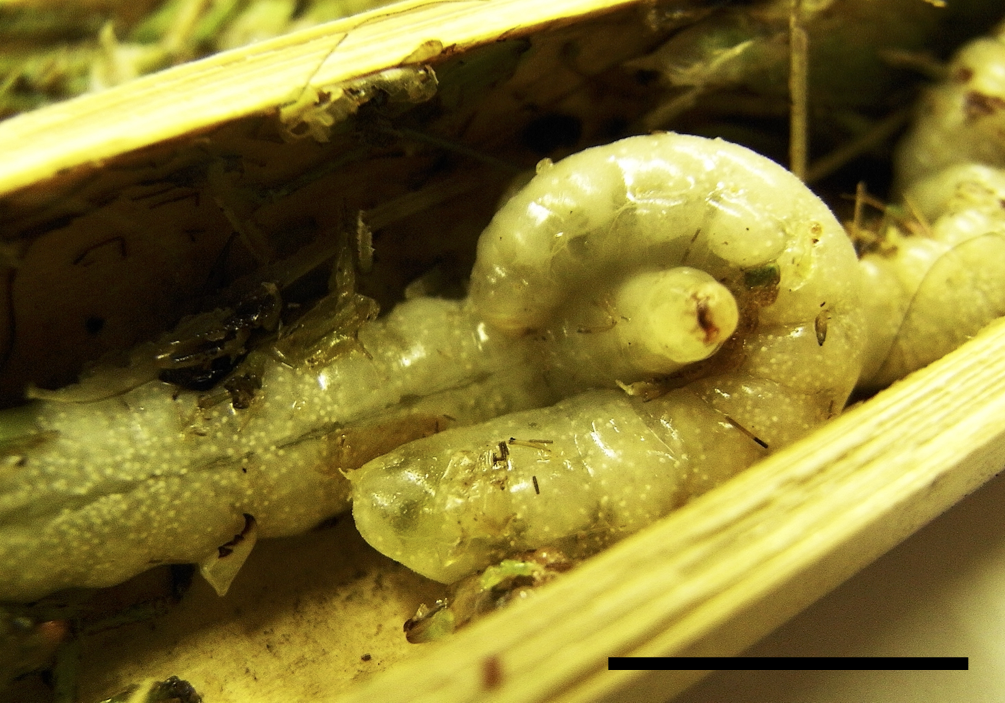

Supplement: S8 Fig — A larva bending its body like mirror writing of a letter “J” was biting another larva at the thoracic part. Body lengths of both cannibal and its victim were around 20 mm. Scale bar indicates 10 mm at the center of the photo. Photo was taken just after opening the nest built in the bamboo cane trap by YI. Nest was sampled at August 8, 2010. Nest no. 65. (TIF) [file pone.0267958.s008.tif]
