# Supplementary material for: Brood reduction caused by sibling cannibalism in Isodontia harmandi (Hymenoptera: Sphecidae), a solitary wasp species building communal brood cells
Source: PLoS One. 2022 May 18;17(5):e0267958. doi: 10.1371/journal.pone.0267958 (PMC9116661; doi:10.1371/journal.pone.0267958)
Supplement: S5 File — (DOCX) [file pone.0267958.s013.docx]

S5 File. Results of statistical analyses.

Table A. Results of GLMM for clutch size of *Isodontia harmandi* nests.

| Variable | Level | Estimate | SE | z | p |
| --- | --- | --- | --- | --- | --- |
| GLMM for clutch size (strict data set) | | |  |  |  |
| Intercept |  | 2.22 | 0.15 | 15.05 | <0.001 *** |
| Brood sex | Male brood | -0.24 | 0.17 | -1.39 | 0.17 |
|  | Sex-unknown brood | -0.31 | 0.2 | -1.57 | 0.12 |
| Multiple comparison test for brood-sex categories | | | |  |  |
| Male vs. Female | | -0.24 | 0.17 | -1.39 | 0.34 |
| Male vs. Female | | -0.31 | 0.2 | -1.57 | 0.26 |
| Male vs. Female | | -0.07 | 0.15 | -0.45 | 0.89 |
|  | | | | | |
| GLMM for clutch size (broad data set) | | | | | |
| Intercept |  | 2.12 | 0.12 | 17.95 | <0.001 *** |
| Brood-sex | Male brood | -0.12 | 0.14 | -0.86 | 0.39 |
|  | Sex-unknown brood | -0.18 | 0.18 | -0.98 | 0.33 |
| Multiple comparison test for brood-sex categories | | | | | |
| Male vs. Female | | -0.12 | 0.13 | -0.86 | 0.66 |
| Sex-unknown vs. Female | | -0.18 | 0.18 | -0.98 | 0.59 |
| Sex-unknown vs. Male | | -0.06 | 0.16 | -0.37 | 0.93 |

Brood size (=clutch size) was set as the response variables, and brood-sex was set as the explanatory variable, using generalized linear model with a Poisson distribution. Different year data were included as a random factor. Multiple comparison tests were applied to brood-sex categories by Tukey method.

Table B. Results of GLMM for the number of cocoons in *Isodontia harmandi* nests.

| Variable | Level | Estimate | SE | z | p |
| --- | --- | --- | --- | --- | --- |
| GLMM for cocoon number | | | | | |
| Intercept |  | 1.43 | 0.12 | 12.05 | <0.001 *** |
| Brood sex | Male brood | 0.02 | 0.14 | 0.18 | 0.86 |
|  | Sex-unknown brood | -0.07 | 0.15 | -0.45 | 0.66 |
| Multiple comparison test for brood-sex categories | | | | | |
| Male vs. Female | | 0.02 | 0.14 | 0.18 | 0.98 |
| Sex-unknown vs. Female | | -0.07 | 0.15 | -0.45 | 0.90 |
| Sex-unknown vs. Male | | -0.09 | 0.12 | -0.78 | 0.72 |

Number of cocoons in *Isodontia harmandi* nests was set as the response variable and brood-sex categories was set as the explanatory variable for generalized liner model, using a Poisson distribution. Different year data were included as a random factor. Multiple comparison tests were applied to brood-sex categories by Tukey method.

Table C. Results of GLMM for brood size in overall *Isodontia harmandi* nests.

| Variable | Level | | | Estimate | | | SE | | | z | | | p | | |
| --- | --- | --- | --- | --- | --- | --- | --- | --- | --- | --- | --- | --- | --- | --- | --- |
| GLMM for brood size | | | | | | | | | | | | | | | |
| Intercept |  | | | 1.86 | | | 0.13 | | | 14.43 | | | <0.001 *** | | |
| Stage | 2 | | | -0.02 | | | 0.11 | | | -0.15 | | | 0.88 | | |
|  | 3 | | | -0.35 | | | 0.12 | | | -2.83 | | | 0.004 ** | | |
|  | 4 | | | -0.42 | | | 0.14 | | | -3.07 | | | 0.002 ** | | |
|  | 5 | | | -0.61 | | | 0.13 | | | -4.57 | | | <0.001 *** | | |
| Phase | 3 | | | 0.25 | | | 0.13 | | | 1.94 | | | 0.05 * | | |
|  | 4 | | | 0.28 | | | 0.15 | | | 1.82 | | | 0.07 | | |
|  | 5 | | | 0.28 | | | 0.15 | | | 1.89 | | | 0.06 | | |
| Brood-sex | Male | | | -0.10 | | | 0.08 | | | -1.32 | | | 0.19 | | |
|  | Sex-unknown | | | -0.25 | | | 0.08 | | | -3.1 | | | 0.002 ** | | |
| Multiple comparison test for developmental stages | | | | | | | | | | | | | | | |
| Stage 2 vs. 1 | | | | 0.02 | | | 0.11 | | | 0.15 | | | 1.00 | | |
| Stage 3 vs. 1 | | | | 0.35 | | | 0.12 | | | 2.83 | | | 0.03 * | | |
| Stage 4 vs. 1 | | | | 0.42 | | | 0.14 | | | 3.07 | | | 0.02 * | | |
| Stage 5 vs. 1 | | | | 0.61 | | | 0.13 | | | 4.57 | | | <0.001 *** | | |
| Stage 3 vs. 2 | | | | 0.33 | | | 0.07 | | | 4.63 | | | <0.001 *** | | |
| Stage 4 vs. 2 | | | | 0.40 | | | 0.09 | | | 4.68 | | | <0.001 *** | | |
| Stage 5 vs. 2 | | | | 0.59 | | | 0.08 | | | 7.36 | | | <0.001 *** | | |
| Stage 4 vs. 3 | | | | 0.07 | | | 0.08 | | | 0.81 | | | 0.92 | | |
| Stage 5 vs. 3 | | | | 0.26 | | | 0.07 | | | 3.45 | | | 0.004 ** | | |
| Stage 5 vs. 4 | | | | 0.19 | | | 0.08 | | | 2.36 | | | 0.12 | | |
| Multiple comparison test for nesting phases | | | | | | | | | | | | | | | |
| Phase 3 vs. 2 | | | | 0.25 | | | 0.13 | | | 1.94 | | | 0.20 | | |
| Phase 4 vs. 2 | | | | 0.28 | | | 0.15 | | | 1.82 | | | 0.25 | | |
| Phase 5 vs. 2 | | | | 0.28 | | | 0.15 | | | 1.89 | | | 0.22 | | |
| Phase 4 vs. 3 | | | | 0.03 | | | 0.09 | | | 0.29 | | | 0.99 | | |
| Phase 5 vs. 3 | | | | 0.02 | | | 0.08 | | | 0.30 | | | 0.99 | | |
| Phase 5 vs. 4 | | | | -0.003 | | | 0.08 | | | -0.04 | | | 1.00 | | |
| Multiple comparison test for brood-sex categories | | | | | | | | | | | | | | | |
| Male vs. Female | | | | -0.10 | | | 0.08 | | | -1.32 | | | 0.38 | | |
| Sex-unknown vs. Female | | | | -0.25 | | | 0.08 | | | -3.10 | | | 0.005 ** | | |
| Sex-unknown vs. Male | | | | -0.15 | | | 0.06 | | | -2.63 | | | 0.02 * | | |
|  | | | | | | | | | | | | | | | |
| GLMM for female brood size | | | | | | | | | | | | | | | |
| Intercept | |  | | | 2.34 | | | 0.33 | | | 6.99 | | | <0.001 *** | |
| Stage | | 2 | | | -0.31 | | | 0.37 | | | -0.85 | | | 0.40 | |
|  | | 3 | | | -0.56 | | | 0.40 | | | -1.41 | | | 0.16 | |
|  | | 4 | | | -0.85 | | | 0.48 | | | -1.80 | | | 0.07 | |
|  | | 5 | | | -1.02 | | | 0.47 | | | -2.18 | | | 0.03 * | |
| Phase | | 4 | | | 0.18 | | | 0.22 | | | 0.81 | | | 0.42 | |
|  | | 5 | | | 0.04 | | | 0.24 | | | 0.17 | | | 0.86 | |
| Multiple comparison test for developmental stages | | | | | | | | | | | | | | | |
| Stage 2 vs. 1 | | | | | -0.31 | | | 0.37 | | | -0.85 | | | 0.90 | |
| Stage 3 vs. 1 | | | | | -0.56 | | | 0.40 | | | -1.41 | | | 0.59 | |
| Stage 4 vs. 1 | | | | | -0.85 | | | 0.48 | | | -1.80 | | | 0.35 | |
| Stage 5 vs. 1 | | | | | -1.02 | | | 0.47 | | | -2.18 | | | 0.17 | |
| Stage 3 vs. 2 | | | | | -0.25 | | | 0.19 | | | -1.32 | | | 0.65 | |
| Stage 4 vs. 2 | | | | | -0.54 | | | 0.31 | | | -1.74 | | | 0.38 | |
| Stage 5 vs. 2 | | | | | -0.71 | | | 0.27 | | | -2.61 | | | 0.06 | |
| Stage 4 vs. 3 | | | | | -0.30 | | | 0.30 | | | -0.98 | | | 0.85 | |
| Stage 5 vs. 3 | | | | | -0.46 | | | 0.25 | | | -1.86 | | | 0.31 | |
| Stage 5 vs. 4 | | | | | -0.16 | | | 0.27 | | | -0.60 | | | 0.97 | |
| Multiple comparison test for nesting phases | | | | | | | | | | | | | | | |
| Phase 4 vs. 3 | | | | | 0.18 | | | 0.22 | | | 0.81 | | | 0.70 | |
| Phase 5 vs. 3 | | | | | 0.04 | | | 0.24 | | | 0.17 | | | 0.98 | |
| Phase 5 vs. 4 | | | | | -0.14 | | | 0.24 | | | -0.58 | | | 0.83 | |
| Level  Estimate  SE  z  p | | | | | | | | | | | | | | | |
| GLMM for male brood size | | | | | | | | | | | | | | | |
| Intercept | | |  | | 1.93 | | | 0.21 | | | 9.24 | | | <0.001 *** | |
| Stage | | | 2 | | 0.02 | | | 0.18 | | | 0.13 | | | 0.89 | |
|  | | | 3 | | -0.23 | | | 0.18 | | | -1.25 | | | 0.21 | |
|  | | | 4 | | -0.30 | | | 0.20 | | | -1.48 | | | 0.14 | |
|  | | | 5 | | -0.52 | | | 0.20 | | | -2.62 | | | 0.009 ** | |
| Phase | | | 3 | | 0.05 | | | 0.21 | | | 0.21 | | | 0.83 | |
|  | | | 4 | | -0.01 | | | 0.23 | | | -0.02 | | | 0.98 | |
|  | | | 5 | | -0.01 | | | 0.22 | | | -0.02 | | | 0.98 | |
| Multiple comparison test for developmental stage | | | | | | | | | | | | | | | |
| Stage 2 vs. 1 | | | | | 0.02 | | | 0.18 | | | 0.14 | | | 1.00 | |
| Stage 3 vs. 1 | | | | | 0.23 | | | 0.18 | | | -1.26 | | | 0.70 | |
| Stage 4 vs. 1 | | | | | 0.30 | | | 0.20 | | | -1.48 | | | 0.56 | |
| Stage 5 vs. 1 | | | | | 0.52 | | | 0.20 | | | -2.62 | | | 0.06 | |
| Stage 3 vs. 2 | | | | | 0.26 | | | 0.10 | | | -2.58 | | | 0.07 | |
| Stage 4 vs. 2 | | | | | 0.33 | | | 0.12 | | | -2.70 | | | 0.05 * | |
| Stage 5 vs. 2 | | | | | 0.54 | | | 0.11 | | | -4.88 | | | <0.001 *** | |
| Stage 4 vs. 3 | | | | | 0.07 | | | 0.11 | | | -0.63 | | | 0.97 | |
| Stage 5 vs. 3 | | | | | 0.29 | | | 0.10 | | | -2.84 | | | 0.03 * | |
| Stage 5 vs. 4 | | | | | 0.22 | | | 0.12 | | | -1.84 | | | 0.33 | |
| Multiple comparison test for nesting phase | | | | | | | | | | | | | | | |
| Phase 3 vs. 2 | | | | | 0.05 | | | 0.21 | | | 0.21 | | | 1.00 | |
| Phase 4 vs. 2 | | | | | -0.01 | | | 0.23 | | | -0.02 | | | 1.00 | |
| Phase 5 vs. 2 | | | | | -0.01 | | | 0.22 | | | -0.02 | | | 1.00 | |
| Phase 4 vs. 3 | | | | | -0.05 | | | 0.13 | | | -0.37 | | | 0.98 | |
| Phase 5 vs. 3 | | | | | -0.05 | | | 0.12 | | | -0.43 | | | 0.97 | |
| Phase 5 vs. 4 | | | | | 0.00 | | | 0.10 | | | 0.01 | | | 1.00 | |
|  | | | | | | | | | | | | | | | |
| GLMM for sex-unknown brood size | | | | | | | | | | | | | | | |
| (Intercept) | | |  | | | 1.55 | | | 0.12 | | | 12.75 | | | <0.001 *** |
| Stage | | | 2 | | | -0.05 | | | 0.17 | | | -0.30 | | | 0.77 |
|  | | | 3 | | | -0.58 | | | 0.20 | | | -2.91 | | | 0.004 ** |
|  | | | 4 | | | -0.53 | | | 0.21 | | | -2.57 | | | 0.01 * |
|  | | | 5 | | | -0.69 | | | 0.21 | | | -3.29 | | | 0.001 ** |
| Phase | | | 3 | | | 0.35 | | | 0.18 | | | 1.99 | | | 0.05 * |
|  | | | 4 | | | 0.38 | | | 0.25 | | | 1.54 | | | 0.12 |
|  | | | 5 | | | 0.49 | | | 0.21 | | | 2.33 | | | 0.02 * |
| Multiple comparison test for developmental stages | | | | | | | | | | | | | | | |
| Stage 2 vs. 1 | | | | | | 0.05 | | | 0.17 | | | -0.30 | | | 1.00 |
| Stage 3 vs. 1 | | | | | | 0.58 | | | 0.20 | | | -2.91 | | | 0.03 * |
| Stage 4 vs. 1 | | | | | | 0.53 | | | 0.21 | | | -2.57 | | | 0.07 |
| Stage 5 vs. 1 | | | | | | 0.69 | | | 0.21 | | | -3.29 | | | 0.008 ** |
| Stage 3 vs. 2 | | | | | | 0.53 | | | 0.13 | | | -3.92 | | | <0.001 *** |
| Stage 4 vs. 2 | | | | | | 0.48 | | | 0.14 | | | -3.44 | | | 0.005 ** |
| Stage 5 vs. 2 | | | | | | 0.64 | | | 0.14 | | | -4.48 | | | <0.001 *** |
| Stage 4 vs. 3 | | | | | | 0.04 | | | 0.13 | | | 0.33 | | | 1.00 |
| Stage 5 vs. 3 | | | | | | 0.11 | | | 0.13 | | | -0.82 | | | 0.92 |
| Stage 5 vs. 4 | | | | | | 0.16 | | | 0.13 | | | -1.24 | | | 0.72 |
| Multiple comparison test for nesting phases | | | | | | | | | | | | | | | |
| Phase 3 vs. 2 | | | | | | 0.35 | | | 0.18 | | | 1.99 | | | 0.18 |
| Phase 4 vs. 2 | | | | | | 0.38 | | | 0.25 | | | 1.54 | | | 0.39 |
| Phase 5 vs. 2 | | | | | | 0.49 | | | 0.21 | | | 2.33 | | | 0.08 |
| Phase 4 vs. 3 | | | | | | 0.03 | | | 0.18 | | | 0.16 | | | 1.00 |
| Phase 5 vs. 3 | | | | | | 0.15 | | | 0.13 | | | 1.09 | | | 0.68 |
| Phase 5 vs. 4 | | | | | | 0.12 | | | 0.16 | | | 0.74 | | | 0.87 |

Brood size of *Isodontia harmandi* nests was set as the response variable and developmental stage, nesting phase, and brood-sex were set as the explanatory variables for generalized linear mixed model, using a Poisson distribution. Different year data were included as a random factor. Multiple comparison tests were applied to all explanatory variables by Tukey method.

Table D. Results of GLMM for prey weight of *Isodontia harmandi* nests among brood-sex categories.

| Variable | Level | Estimate | SE | z | p |
| --- | --- | --- | --- | --- | --- |
| GLMM for prey weight | | | | | |
| Intercept |  | 3.74 | 0.42 | 8.99 | <0.001 *** |
| Brood-sex | Male | -0.88 | 0.39 | -2.23 | 0.03 * |
|  | Sex-unknown | -1.04 | 0.44 | -2.39 | 0.02 * |
| Stage |  | 0.18 | 0.27 | 0.68 | 0.50 |
| Phase |  | 0.02 | 0.31 | 0.07 | 0.94 |
| Multiple comparison test for brood-sex categories | | | | | |
| Male vs. Female | | -0.87 | 0.39 | -2.23 | 0.06 |
| Sex-unknown vs. Female | | -1.04 | 0.44 | -2.39 | 0.04 * |
| Sex-unknown vs. Male | | -0.16 | 0.31 | -0.54 | 0.84 |

Total prey weight of nests was set as the response variable and brood-sex, developmental stage and nesting phase were set as the explanatory variables for generalized linear mixed model, using a Gaussian distribution. Different years data were included as a random factor. Multiple comparison test was applied to brood-sex categories by Tukey method.

Table E. Results of GLMM for brood size effect on total prey weight in overall *Isodontia harmandi* nests.

| Variable | Level | Estimate | SE | z | p |
| --- | --- | --- | --- | --- | --- |
| GLMM for brood size effects to prey weight | | | | | |
| Intercept |  | 2.76 | 0.99 | 2.79 | <0.001 *** |
| Brood size |  | 0.08 | 0.09 | 0.93 | 0.36 |
| Brood-sex | Male | -0.47 | 0.59 | -0.79 | 0.44 |
|  | Sex-unknown | -0.58 | 0.68 | -0.86 | 0.40 |
| Multiple comparison test for brood-sex categories | | | | | |
| Male vs. Female | | -0.46 | 0.59 | -0.79 | 0.70 |
| Sex-unknown vs. Female | | -0.58 | 0.68 | -0.86 | 0.66 |
| Sex-unknown vs. Male | | -0.11 | 0.48 | -0.23 | 0.97 |
|  | | | | | |
| GLM for brood size effect to prey weight in female brood | | | | | |
| Intercept | | 3.75 | 0.68 | 5.49 | 0.01 * |
| Brood size | | -0.02 | 0.07 | -0.30 | 0.78 |
|  | | | | | |
| GLMM for brood size effect to prey weight in male brood | | | | | |
| Intercept | | 2.27 | 0.99 | 2.29 | 0.04 * |
| Brood size | | 0.09 | 0.13 | 0.69 | 0.50 |
|  | | | | | |
| GLMM for brood size effect to prey weight in sex-unknown brood | | | | | |
| Intercept | | 2.00 | 1.55 | 1.29 | 0.25 |
| Brood size | | 0.13 | 0.22 | 0.61 | 0.57 |

Total prey weight per brood (g) was set as the response variable and brood size and brood-sex were set as the explanatory variables for generalized linear mixed model, using a Gaussian distribution. Different years data were included as a random factor. Multiple comparison test was applied to brood-sex categories by Tukey method. Data used in the analysis was the same as the strict one for clutch size. For female broods, general linear model was used.

Table F. Results of GLMM for brood size effect on per-capita prey weight in overall *Isodontia harmandi* nests.

| Variable | Level | Estimate | SE | z | p |
| --- | --- | --- | --- | --- | --- |
| GLMM for brood size effects to per-capita prey weight | | | | | |
| Intercept |  | 1.11 | 0.17 | 6.44 | <0.001 *** |
| Brood size |  | -0.07 | 0.02 | -4.63 | <0.001 *** |
| Brood sex | Male | -0.14 | 0.11 | -1.30 | 0.20 |
|  | Sex-unknown | -0.10 | 0.12 | -0.84 | 0.41 |
| Multiple comparison test for brood-sex categories | | | | | |
| Male vs. Female | | -0.14 | 0.11 | -1.31 | 0.39 |
| Sex-unknown vs. Female | | -0.10 | 0.12 | -0.85 | 0.67 |
| Sex-unknown vs. Male | | 0.04 | 0.08 | 0.46 | 0.89 |
|  | | | | | |
| GLM for brood size effect to per-capita prey weight in female brood | | | | | |
| Intercept | | 0.97 | 0.13 | 7.62 | 0.005 ** |
| Brood size | | -0.06 | 0.01 | -4.41 | 0.02 * |
|  | | | | | |
| GLMM for brood size effect to per-capita prey weight in male brood | | | | | |
| Intercept | | 0.85 | 0.16 | 5.49 | <0.001 *** |
| Brood size | | -0.06 | 0.02 | -2.79 | 0.02 * |
|  | | | | | |
| GLMM for brood size effect to per-capita prey weight in sex-unknown brood | | | | | |
| Intercept | | 1.35 | 0.28 | 4.87 | 0.005 ** |
| Brood size | | -0.12 | 0.04 | -3.17 | 0.02 * |

Per-capita prey weight (g) was set as the response variable and brood size and brood-sex were set as the explanatory variables for generalized linear mixed model, using a Gaussian distribution. Different years data were included as a random factor. Multiple comparison test was applied to brood-sex categories by Tukey method. Data used in the analysis was the same as the strict one for clutch size. For female broods, general linear model was used.

Table G. Results of GLMM for larval survival of *Isodontia harmandi* nests in whole brood rearing experiment.

| Variable | Level | Estimate | SE | z | p |
| --- | --- | --- | --- | --- | --- |
| GLMM for larval survival | | | | | |
| Intercept |  | 2.10 | 1.70 | 1.24 | 0.22 |
| Prey weight |  | 0.59 | 0.30 | 1.97 | 0.05 * |
| No eggs & larvae (Brood size) | | -0.35 | 0.12 | -2.83 | 0.005 ** |
| Brood-sex | Male | -0.48 | 0.83 | -0.57 | 0.57 |
|  | Sex-unknown | -2.53 | 1.21 | -2.09 | 0.04 * |
| Multiple comparison test for brood-sex categories | | | | | |
| Male vs. Female | | -0.48 | 0.83 | -0.57 | 0.82 |
| Sex-unknown vs. Female | | -2.53 | 1.21 | -2.09 | 0.08 |
| Sex-unknown vs. Male | | -2.06 | 0.80 | -2.56 | 0.03 * |

Larval survival (number of offspring survived to cocoon stage and number of offspring died until cocoon stage within a brood) was set as the response variable and total prey weight, number of eggs and larvae (brood size) and brood-sex were set as the explanatory variables for generalized linear mixed model, using a binomial distribution. Different years (2010 and 2015) data and nest identification were included as random factors. Multiple comparison test was applied to brood-sex categories by Tukey method. Whole-brood rearing data were used in the analysis.

Table H. Results of GLMM analysis for cocoon survival of *Isodontia harmandi* nests in whole brood rearing experiment.

| Variable | Level | Estimate | SE | z | p |
| --- | --- | --- | --- | --- | --- |
| GLMM for cocoon survival | | | | | |
| Intercept |  | -0.21 | 1.16 | -0.18 | 0.86 |
| Prey weight |  | 0.08 | 0.24 | 0.33 | 0.74 |
| No. of eggs & larvae (Brood size) | | 0.05 | 0.12 | 0.40 | 0.69 |
| Brood-sex | Male | 0.46 | 0.54 | 0.86 | 0.39 |
|  | Sex-unknown | 0.03 | 0.92 | 0.03 | 0.98 |
| Multiple comparison test for brood-sex categories | | | | | |
| Male vs. Female | | 0.46 | 0.54 | 0.86 | 0.65 |
| Sex-unknown vs. Female | | 0.03 | 0.92 | 0.03 | 1.00 |
| Sex-unknown vs. Male | | -0.44 | 0.79 | -0.56 | 0.84 |

Cocoon survival (the number of offspring successfully emerging as adult stage and the number of offspring died during cocoon and adult stage within a brood) was set as the response variable and total prey weight, number of eggs and larvae (brood size) and brood-sex was set as the explanatory variable for general linear model, using a binomial distribution. Different years (2010 and 2015) data and nest identification were included as random factors. Multiple comparison test was applied to brood-sex categories by Tukey method. Data of survived cocoons extracted from whole-brood rearing data were used in the analysis (n=7 for female brood, n=21 for male broods, and n=5 for sex-unknown broods).
