# Supplementary material for: Brood reduction caused by sibling cannibalism in Isodontia harmandi (Hymenoptera: Sphecidae), a solitary wasp species building communal brood cells
Source: PLoS One. 2022 May 18;17(5):e0267958. doi: 10.1371/journal.pone.0267958 (PMC9116661; doi:10.1371/journal.pone.0267958)
Supplement: S6 File — (PDF) [file pone.0267958.s014.pdf]

Supplementary Information for

Brood reduction caused by sibling cannibalism in *Isodontia harmandi* (Hymenoptera: Sphecidae), a solitary wasp species building communal brood cell

Yui Imasaki* and Tomoji Endo*

* Department of Biosphere Sciences, School of Human Sciences, Kobe College, Nishinomiya, Hyogo, 662-8505, Japan

Contents:

Supplementary Tables S1-S4

Supplementary Figures S1-S6

Supplementary Table S1. Numbers of nests with different developmental stages of brood at different nesting phases in *Isodontia harmandi* nests collected during 2010 and 2015. Numerals are the number of nests. Numerals in parenthesis are the number of female broods, mele broods, and broods which sex were not determined, respectively. The numbers of nests surrounded by dotted lines in each year were used for analyses of brood reduction. Nests in shadow areas were used for determining the total clutch size strictly. Out of nests in half-tone areas, those provisioned with more than 17 prey were used as additional data to estimate the total clutch size.

				Developmental stage of brood							
	Study site	Nesting phase	No egg & no prey	[1] Eggs	[2] Hatchlings	[3] Middle-sized larvae	[4] Full-grown larvae	[5] Cocoons	Attacked or prey rotten	Prey stored but no egg	Total
2010	Minami-yashiro	(i) Pre-hunting	38	0	0	0	0	0	0	0	38
		(ii) Egg-laying	0	6 (0, 0, 6)	0 (0, 0, 0)	0 (0, 0, 0)	0 (0, 0, 0)	0 (0, 0, 0)	1 (0, 0, 1)	0	7 (0, 0, 7)
		(iii) Provisioning	0	0 (0, 0, 0)	11 (1, 3, 7)	8 (1, 3, 4)	0 (0, 0, 0)	0 (0, 0, 0)	13 (0, 1, 12)	0	32 (2, 7, 23)
		(iv) Closing	0	0 (0, 0, 0)	3 (2, 1, 0)	3 (2, 0, 1)	3 (0, 2, 1)	0 (0, 0, 0)	5 (0, 2, 3)	0	14 (4, 5, 5)
		(v) Completed	0	0 (0, 0, 0)	1 (0, 1, 0)	11 (1, 9, 1)	10 (2, 4, 4)	40 (15, 17, 8)	11 (2, 4, 5)	1	74 (20, 35, 18)
	Total		38	6 (0, 0, 6)	15 (3, 5, 7)	22 (4, 12, 6)	13 (2, 6, 5)	40 (15, 17, 8)	30 (2, 7, 21)	1	165 (26, 47, 53)
2011	Minami-yashiro	(i) Pre-hunting	10	0	0	0	0	0	0	0	10 (0, 0, 0)
		(ii) Egg-laying	0	2 (0, 0, 2)	1 (0, 1, 0)	0 (0, 0, 0)	0 (0, 0, 0)	0 (0, 0, 0)	0 (0, 0, 0)	0	3 (0, 1, 2)
		(iii) Provisioning	0	8 (1, 3, 4)	4 (2, 1, 1)	1 (1, 0, 0)	0 (0, 0, 0)	0 (0, 0, 0)	1 (0, 1, 0)	0	14 (4, 5, 5)
		(iv) Closing	0	0 (0, 0, 0)	1 (0, 0, 1)	2 (0, 1, 1)	0 (0, 0, 0)	0 (0, 0, 0)	0 (0, 0, 0)	0	3 (0, 1, 2)
		(v) Completed	0	0 (0, 0, 0)	12 (1, 5, 6)	6 (0, 4, 2)	7 (2, 1, 4)	1 (0, 0, 1)	0 (0, 0, 0)	1	27 (3, 10, 13)
	Total		10	10 (1, 3, 6)	18 (3, 7, 8)	9 (1, 5, 3)	7 (2, 1, 4)	1 (0, 0, 1)	1 (0, 1, 0)	1	57 (7, 17, 22)
2012	Furuichi	(i) Pre-hunting	5	0	0	0	0	0	0	0	5 (0, 0, 0)
		(ii) Egg-laying	0	1 (0, 0, 1)	1 (0, 1, 0)	0 (0, 0, 0)	0 (0, 0, 0)	0 (0, 0, 0)	2 (0, 0, 2)	1	5 (0, 1, 3)
		(iii) Provisioning	0	3 (0, 1, 2)	4 (1, 1, 2)	1 (0, 1, 0)	0 (0, 0, 0)	0 (0, 0, 0)	0 (0, 0, 0)	0	8 (1, 3, 4)
		(iv) Closing	0	0 (0, 0, 0)	0 (0, 0, 0)	1 (0, 1, 0)	0 (0, 0, 0)	0 (0, 0, 0)	1 (1, 0, 0)	0	2 (1, 1, 0)
		(v) Completed	0	0 (0, 0, 0)	7 (1, 5, 1)	14 (2, 3, 9)	5 (0, 0, 5)	6 (0, 0, 6)	3 (0, 1, 2)	0	35 (3, 9, 23)
	Total		5	4 (0, 1, 3)	12 (2, 7, 3)	16 (2, 5, 9)	5 (0, 0, 5)	6 (0, 0, 6)	6 (1, 1, 4)	1	55 (5, 14, 30)
2013	Kusano	(i) Pre-hunting	26	0	0	0	0	0	0	0	26 (0, 0, 0)
		(ii) Egg-laying	0	2 (0, 1, 1)	1 (0, 0, 1)	0 (0, 0, 0)	0 (0, 0, 0)	0 (0, 0, 0)	0 (0, 0, 0)	1	4 (0, 1, 2)
		(iii) Provisioning	0	0 (0, 0, 0)	2 (0, 1, 1)	5 (0, 4, 1)	1 (0, 0, 1)	0 (0, 0, 0)	1 (0, 1, 0)	0	9 (0, 6, 3)
		(iv) Closing	0	0 (0, 0, 0)	5 (1, 4, 0)	7 (1, 4, 2)	5 (0, 4, 1)	4 (0, 4, 0)	7 (0, 1, 6)	0	28 (2, 17, 9)
		(v) Completed	0	0 (0, 0, 0)	1 (0, 1, 0)	15 (0, 12, 3)	22 (0, 13, 9)	28 (2, 20, 6)	25 (1, 3, 21)	3	94 (3, 49, 39)
	Total		26	2 (0, 1, 1)	9 (1, 6, 2)	27 (1, 20, 6)	28 (0, 17, 11)	32 (2, 24, 6)	33 (1, 5, 27)	4	161 (5, 73, 53)
2015	Kirihata	(i) Pre-hunting	9	0	0	0	0	0	0	0	9 (0, 0, 0)
		(ii) Egg-laying	0	4 (0, 1, 3)	1 (0, 0, 1)	0 (0, 0, 0)	0 (0, 0, 0)	0 (0, 0, 0)	3 (0, 0, 3)	0	8 (0, 1, 7)
		(iii) Provisioning	0	1 (0, 1, 0)	4 (0, 1, 3)	1 (0, 0, 1)	0 (0, 0, 0)	0 (0, 0, 0)	1 (0, 0, 1)	1	8 (0, 2, 5)
		(iv) Closing	0	0 (0, 0, 0)	3 (0, 2, 1)	0 (0, 0, 0)	2 (0, 0, 2)	2 (0, 1, 1)	3 (0, 0, 3)	0	10 (0, 3, 7)
		(v) Completed	0	0 (0, 0, 0)	0 (0, 0, 0)	2 (0, 1, 1)	1 (0, 0, 1)	15 (0, 2, 13)	4 (0, 0, 4)	0	22 (0, 3, 19)
	Total		9	5 (0, 2, 3)	8 (0, 3, 5)	3 (0, 1, 2)	3 (0, 0, 3)	17 (0, 3, 14)	11 (0, 0, 11)	1	57 (0, 9, 38)
2010-2015		(i) Pre-hunting	88	0	0	0	0	0	0	0	88 (0, 0, 0)
		(ii) Egg-laying	0	15 (0, 2, 13)	4 (0, 2, 2)	0 (0, 0, 0)	0 (0, 0, 0)	0 (0, 0, 0)	6 (0, 0, 6)	2	27 (0, 4, 21)
		(iii) Provisioning	0	12 (1, 5, 6)	25 (4, 7, 14)	16 (2, 8, 6)	1 (0, 0, 1)	0 (0, 0, 0)	16 (0, 3, 13)	1	71 (7, 23, 40)
		(iv) Closing	0	0 (0, 0, 0)	12 (3, 7, 2)	13 (3, 6, 4)	10 (0, 6, 4)	6 (0, 5, 1)	16 (1, 3, 12)	0	57 (7, 27, 23)
		(v) Completed	0	0 (0, 0, 0)	21 (2, 12, 7)	48 (3, 29, 16)	45 (4, 18, 23)	90 (17, 39, 34)	43 (3, 8, 32)	5	252 (29, 106, 112)
	Total		88	27 (1, 7, 19)	62 (9, 28, 25)	77 (8, 43, 26)	56 (4, 24, 28)	96 (17, 44, 35)	81 (4, 14, 63)	8	495 (43, 160, 196)

Supplementary Table S2-1. Seasonal changes in mean body weight (g) of prey item for four prey species provisoned into the nests of *Isodontia harmandi* during 2010-2015. Figures are given as sample size (n), mean and standard deviation (sd) of body weight for both sexes of prey items which have not been consumed in the nest collected on each sampling day.

		Cosmetura fenestrata											
Sampling		Female			Male			Unknown			Total		
Year	Date	n	mean	sd	n	mean	sd	n	mean	sd	n	mean	sd
2010	7/23	61	0.082	0.021	26	0.070	0.014	3	0.057	0.031	90	0.077	0.020
	7/27	121	0.095	0.017	119	0.076	0.021	0			240	0.086	0.021
	8/5	11	0.141	0.078	7	0.080	0.023	0			18	0.117	0.069
	8/10	8	0.102	0.034	4	0.091	0.038	0			12	0.099	0.034
	8/26	0			0			0			0		
2010 total		201	0.094	0.029	156	0.076	0.020	3	0.057	0.031	360	0.086	0.027
2011	7/26	3	0.113	0.012	2	0.075	0.007	6	0.100	0.023	11	0.099	0.022
	8/4	97	0.108	0.014	68	0.085	0.013	1	0.040	-	166	0.098	0.018
	8/6	17	0.133	0.040	11	0.117	0.024	6	0.100	0.026	34	0.122	0.035
	8/8	78	0.100	0.014	45	0.081	0.011	0			123	0.093	0.016
	8/10	42	0.104	0.019	32	0.075	0.013	0			74	0.092	0.022
	8/13	43	0.099	0.015	57	0.076	0.016	0			100	0.086	0.019
	8/17	13	0.095	0.015	14	0.083	0.010	0			27	0.089	0.014
	8/20	9	0.099	0.020	22	0.076	0.013	0			31	0.083	0.018
	8/25	3	0.093	0.025	2	0.085	0.007	0			5	0.090	0.019
	8/30	1	0.090	-	2	0.065	0.007	0			3	0.073	0.015
	9/6				0			0			0		
2011 total		306	0.104	0.019	255	0.082	0.016	13	0.095	0.028	574	0.094	0.021
2012	7/26	23	0.108	0.013	24	0.085	0.013	0			47	0.096	0.017
	7/30	38	0.104	0.016	46	0.083	0.018	1	0.080	-	85	0.093	0.020
	8/2	34	0.101	0.016	18	0.077	0.012	0			52	0.093	0.019
	8/5	12	0.105	0.016	33	0.074	0.013	0			45	0.082	0.019
	8/10	8	0.096	0.013	19	0.073	0.012	0			27	0.080	0.017
	8/15	0			7	0.087	0.005	0			7	0.087	0.005
	8/20	1	0.030	-	0			0			1	0.030	-
2012 total		116	0.103	0.017	147	0.079	0.015	1	0.080	-	264	0.090	0.020
2013	7/17	4	0.075	0.016	2	0.074	0.002	0			6	0.075	0.013
	7/20	5	0.089	0.017	2	0.082	0.007	0			7	0.087	0.015
	7/22	51	0.079	0.017	45	0.072	0.009	0			96	0.075	0.014
	7/25	0						0					
	7/29	16	0.081	0.012	15	0.067	0.013	0			31	0.074	0.014
	8/2	3	0.091	0.006				0			3	0.091	0.006
	8/5	10	0.084	0.007	10	0.061	0.009	0			20	0.072	0.014
	8/10	10	0.092	0.010	6	0.075	0.009	0			16	0.085	0.013
	8/13	1	0.026	-				0			1	0.026	-
	8/16	0			1	0.061	-	0			1	0.061	-
	8/22	1	0.046	-	1	0.034	-	0			2	0.040	0.009
2013 total		101	0.081	0.016	82	0.069	0.011	0			183	0.076	0.015
2015	7/25	33	0.111	0.015	25	0.090	0.011	2	0.032	0.010	60	0.100	0.021
	7/30	34	0.111	0.022	16	0.086	0.013	10	0.086	0.033	60	0.100	0.025
	7/31	19	0.100	0.008	25	0.092	0.015	1	0.082	-	45	0.095	0.013
	8/4	5	0.106	0.024	9	0.081	0.009	4	0.064	0.022	18	0.084	0.022
	8/7	11	0.086	0.010	15	0.083	0.015	0			26	0.084	0.013
	8/15	0			0			0			0		
	8/22	0			0			0			0		
2015 total		102	0.106	0.019	90	0.088	0.013	17	0.074	0.033	209	0.096	0.021
Total		826	0.099	0.023	730	0.079	0.017	34	0.081	0.032	1590	0.090	0.023

Supplementary Table S2-2. (continued)

		Hexacentrus hareyamai											
Sampling		Female			Male			Unknown			Total		
Year	Date	n	mean	sd	n	mean	sd	n	mean	sd	n	mean	sd
2010	7/23	0			0			0			0		
	7/27	19	0.187	0.092	38	0.109	0.066	4	0.035	0.022	61	0.129	0.085
	8/5	13	0.199	0.087	11	0.198	0.092	0			24	0.198	0.087
	8/10	6	0.101	0.042	10	0.183	0.087	0			16	0.152	0.083
	8/26	0			0			0			0		
2010 total		38	0.178	0.089	59	0.138	0.084	4	0.035	0.022	101	0.149	0.089
2011	7/26	1	0.120	-	4	0.105	0.039	0			5	0.108	0.034
	8/4	0			0			1	0.050	-	1	0.050	-
	8/6	5	0.294	0.108	6	0.285	0.078	0			11	0.289	0.088
	8/8	0			3	0.270	0.066	0			3	0.270	0.066
	8/10	1	0.320	-	1	0.220	-	0			2	0.270	0.071
	8/13	2	0.210	0.057	0			0			2	0.210	0.057
	8/17	0			0			0			0		
	8/20	0			1	0.250	-	0			1	0.250	-
	8/25	0			0			0			0		
	8/30	0			0			0			0		
	9/6	0			0			0			0		
2011 total		9	0.259	0.102	15	0.227	0.096	1	0.050	-	25	0.232	0.103
2012	7/26	0			0			5	0.108	0.049	5	0.108	0.049
	7/30	6	0.120	0.064	5	0.068	0.024	14	0.086	0.053	25	0.090	0.053
	8/2	0			1	0.070	-	12	0.108	0.044	13	0.105	0.043
	8/5	9	0.144	0.047	12	0.171	0.097	18	0.143	0.046	39	0.152	0.065
	8/10	2	0.295	0.007	0			29	0.192	0.079	31	0.199	0.081
	8/15	0			1	0.320	-	20	0.178	0.084	21	0.185	0.088
	8/20	0			0			0			0		
2012 total		17	0.154	0.073	19	0.146	0.100	98	0.150	0.077	134	0.150	0.079
2013	7/17	1	0.021	-	2	0.019	0.001	0			3	0.020	0.001
	7/20	0			0			1	0.031	-	1	0.031	-
	7/22	8	0.039	0.015	5	0.027	0.008	0			13	0.035	0.014
	7/25	12	0.035	0.017	15	0.036	0.013	0			27	0.036	0.015
	7/29	8	0.028	0.007	3	0.031	0.014	0			11	0.029	0.008
	8/2	19	0.060	0.059	20	0.075	0.063	0			39	0.068	0.061
	8/5	0			2	0.107	0.015	0			2	0.107	0.015
	8/10	15	0.193	0.097	13	0.172	0.110	0			28	0.183	0.102
	8/13	2	0.243	0.135	0			0			2	0.243	0.135
	8/16	0			0			0			0		
	8/22	2	0.217	0.119	4	0.174	0.108	0			6	0.188	0.102
2013 total		67	0.089	0.094	64	0.085	0.086	1	0.031	-	132	0.087	0.089
2015	7/25	0			0			0			0		
	7/30	0			0			0			0		
	7/31	0			0			0			0		
	8/4	0			0			0			0		
	8/7	0			1	0.076	-	0			1	0.076	-
	8/15	0			0			0			0		
	8/22	0			0			0			0		
2015 total		0			1	0.076	-	0			1	0.076	-
Total		131	0.135	0.104	158	0.126	0.096	104	0.144	0.079	393	0.133	0.095

**Supplementary Table S2-3. (continued)**													
		Xiphidiopsis subpunctata											
Sampling		Female			Male			Unknown			Total		
Year	Date	n	mean	sd	n	mean	sd	n	mean	sd	n	mean	sd
2010	7/23	0			0			0			0		
	7/27	0			0			0			0		
	8/5	0			0			0			0		
	8/10	4	0.053	0.003	3	0.057	0.009	0			7	0.055	0.006
	8/26	0			0			0			0		
2010 total		4	0.053	0.003	3	0.057	0.009	0			7	0.055	0.006
2011	7/26	0			0			0			0		
	8/4	0			0			0			0		
	8/6	0			0			0			0		
	8/8	0			1	0.050	-	0			1	0.050	-
	8/10	0			0			0			0		
	8/13	2	0.050	0.000	0			0			2	0.050	0.000
	8/17	1	0.060	-	0			0			1	0.060	-
	8/20	0			1	0.030	-	0			1	0.030	-
	8/25	0			1	0.080	-	0			1	0.080	-
	8/30	0			0			0			0		
	9/6	0			0			0			0		
2011 total		3	0.053	0.006	3	0.053	0.025	0			6	0.053	0.016
2012	7/26	0			0			0			0		
	7/30	0			0			0			0		
	8/2	0			0			0			0		
	8/5	0			0			0			0		
	8/10	0			0			0			0		
	8/15	0			2	0.045	0.007	0			2	0.045	0.007
	8/20	0			0			0			0		
2012 total		0			2	0.045	0.007	0			2	0.045	0.007
2013	7/17	0			0			0			0		
	7/20	0			0			0			0		
	7/22	0			0			0			0		
	7/25	0			0			0			0		
	7/29	0			0			0			0		
	8/2	0			0			0			0		
	8/5	4	0.031	0.007	8	0.040	0.009	0			12	0.037	0.009
	8/10	65	0.046	0.007	61	0.043	0.008	1	0.032	-	127	0.044	0.008
	8/13	9	0.036	0.011	23	0.042	0.014	1	0.031	-	33	0.040	0.013
	8/16	7	0.055	0.008	40	0.051	0.008	0			47	0.052	0.008
	8/22	11	0.050	0.012	22	0.040	0.013	0			33	0.044	0.014
2013 total		96	0.046	0.010	154	0.044	0.011	2	0.032	0.001	252	0.045	0.010
2015	7/25	0			0			0			0		
	7/30	0			0			0			0		
	7/31	0			0			0			0		
	8/4	0			0			0			0		
	8/7	8	0.063	0.010	13	0.067	0.011	0			21	0.066	0.010
	8/15	0			0			6	0.052	0.018	6	0.052	0.018
	8/22	5	0.057	0.018	6	0.049	0.011	0			11	0.052	0.015
2015 total		13	0.061	0.013	19	0.061	0.014	6	0.052	0.018	38	0.060	0.014
Total		116	0.048	0.011	181	0.047	0.012	8	0.047	0.018	305	0.047	0.012

Supplementary Table S2-4. (continued)

		Leptoteratura albicornis												Total		
Sampling		Female			Male			Unknown			Total					
Year	Date	n	mean	sd	n	mean	sd	n	mean	sd	n	mean	sd	n	mean	sd
2010	7/23	0			0			0			0			90	0.077	0.020
	7/27	0			0			0			0			301	0.095	0.046
	8/5	0			1	0.019	-	1	0.014	-	2	0.017	0.003	44	0.157	0.092
	8/10	1	0.038	-	5	0.035	0.009	0			6	0.036	0.008	41	0.103	0.071
	8/26	5	0.044	0.008	3	0.032	0.002	0			8	0.040	0.009	8	0.040	0.009
2010 total		6	0.043	0.007	9	0.032	0.008	1	0.014	-	16	0.035	0.011	484	0.097	0.055
2011	7/26	0			0			0			0			16	0.102	0.025
	8/4	0			0			0			0			167	0.098	0.018
	8/6	0			0			0			0			45	0.163	0.089
	8/8	1	0.020	-	1	0.010	-	0			2	0.015	0.007	129	0.096	0.034
	8/10	0			1	0.040	-	0			1	0.040	-	77	0.096	0.037
	8/13	21	0.043	0.010	10	0.031	0.010	0			31	0.039	0.011	135	0.077	0.031
	8/17	0			0			0			0			28	0.088	0.015
	8/20	80	0.035	0.013	102	0.036	0.009	0			182	0.036	0.011	215	0.043	0.025
	8/25	32	0.039	0.011	61	0.037	0.012	2	0.025	0.007	95	0.037	0.011	101	0.040	0.017
	8/30	41	0.044	0.013	61	0.037	0.012	0			102	0.040	0.013	105	0.040	0.014
	9/6	10	0.043	0.011	16	0.038	0.007	0			26	0.040	0.009	26	0.040	0.009
2011 total		185	0.039	0.013	252	0.036	0.011	2	0.025	0.007	439	0.037	0.012	1044	0.073	0.044
2012	7/26	0			0			0			0			52	0.097	0.022
	7/30	0			0			0			0			110	0.092	0.031
	8/2	0			0			0			0			65	0.095	0.025
	8/5	1	0.060	-	1	0.030	-	6	0.038	0.012	8	0.040	0.013	92	0.108	0.060
	8/10	0			0			8	0.043	0.009	8	0.043	0.009	66	0.131	0.086
	8/15	0			0			25	0.030	0.011	25	0.030	0.011	55	0.097	0.090
	8/20	0			0			0			0			1	0.030	-
2012 total		1	0.060	-	1	0.030	-	39	0.034	0.012	41	0.034	0.012	441	0.103	0.058
2013	7/17	0			0			0			0			9	0.056	0.029
	7/20	0			0			0			0			8	0.080	0.024
	7/22	0			0			0			0			109	0.071	0.019
	7/25	0			0			0			0			27	0.036	0.015
	7/29	0			0			0			0			42	0.062	0.024
	8/2	0			0			0			0			42	0.070	0.059
	8/5	0			1	0.024	-	0			1	0.024	-	35	0.061	0.024
	8/10	0			0			0			0			171	0.071	0.066
	8/13	0			0			0			0			36	0.051	0.054
	8/16	0			1	0.046	-	0			1	0.046	-	49	0.052	0.008
	8/22	0			0			0			0			41	0.065	0.064
2013 total		0			2	0.035	0.015	0			2	0.035	0.015	569	0.064	0.048
2015	7/25	0			0			0			0			60	0.100	0.021
	7/30	0			1	0.098	-	0			1	0.098	-	61	0.100	0.025
	7/31	1	0.091	-	0			1	0.099	-	2	0.095	0.006	47	0.095	0.013
	8/4	0			0			0			0			18	0.084	0.022
	8/7	0			0			0			0			48	0.076	0.015
	8/15	0			0			0			0			6	0.052	0.018
	8/22	0			0			0			0			11	0.052	0.015
2015 total		1	0.091	-	1	0.098	-	1	0.099	-	3	0.096	0.004	251	0.090	0.024
Total		193	0.039	0.013	265	0.036	0.011	43	0.035	0.016	501	0.037	0.012	2789	0.082	0.050

Supplementary Table S3. Yearly average total prey amount (g) of different sex-categories of broods in *Isodontia harmandi* during 2010 and 2015. Different letters indicate statistically significant difference in total prey amount between years.

	Female brood				Male brood				Sex-unknown brood				Overall			
Year	Mean ± SD	Range	(N)		Mean ± SD	Range	(N)		Mean ± SD	Range	(N)		Mean ± SD	Range	(N)	statistics
2010	3.90 ± 0.95	2.96 - 5.10	(5)		2.84 ± 1.36	0.71 - 5.33	(13)		2.16 ± 0.43	1.74 - 2.70	(4)		2.96 ± 1.26	0.71 - 5.33	(22)	ab
2011	3.69	3.69 - 3.69	(1)		2.92 ± 0.70	2.11 - 4.00	(8)		3.28 ± 1.15	0.81 - 4.71	(10)		3.15 ± 0.95	0.81 - 4.71	(19)	a
2012	4.18±0.79	3.45 - 5.01	(3)		3.52 ± 1.34	1.13 - 5.01	(9)		2.73 ± 0.81	1.73 - 3.70	(6)		3.36 ± 1.17	1.13 - 5.01	(18)	a
2013	2.64	2.64 - 2.64	(1)		2.17 ± 1.06	1.28 - 4.15	(10)		1.98 ± 1.18	0.69 - 2.99	(3)		2.16 ± 1.01	0.69 - 4.15	(14)	b
2015	-	-	(0)		3.34 ± 0.62	2.66 - 3.87	(3)		1.53	1.53 - 1.53	(1)		2.89 ± 1.04	1.53 - 3.87	(4)	ab
Total	3.84 ± 0.86	2.64 - 5.1	(10)		2.87 ± 1.20	0.71 - 5.33	(43)		2.72 ± 1.06	0.69 - 4.71	(24)		2.95 ± 1.16	0.69 - 5.33	(77)	

Supplementary Table S4. Outcomes of 21 individual broods in whole brood rearing experiment. Nest nos. were assigned to all nests sampled from study sites.

Year	Locality	Nest No.	Developmental stage of brood*	Nesting phase**	Total prey weight (g)	Initial number of brood (No. egges, No. early larvae)	No. cocoons	No. emerging adults	Sex of brood
2010	Minami-yashiro	4	[3]	(v)	3.21	7 (0, 7)	6	6	Male
2010	Minami-yashiro	5	[3]	(v)	3.65	7 (0, 7)	6	4	Male
2010	Minami-yashiro	10	[3]	(v)	3.60	7 (0, 7)	6	5	Male
2010	Minami-yashiro	11	[2]	(iv)	3.37	8 (0, 8)	7	4	Female
2010	Minami-yashiro	24	[3]	(v)	1.20	3 (0, 3)	3	1	Male
2010	Minami-yashiro	28	[3]	(v)	2.70	7 (0, 7)	7	5	Unknown***
2010	Minami-yashiro	35	[3]	(iv)	5.10	7 (0, 7)	6	5	Female
2010	Minami-yashiro	42	[3]	(v)	3.18	8 (0, 8)	8	2	Male
2010	Minami-yashiro	46	[2]	(iv)	3.35	13 (4, 9)	3	2	Female
2010	Minami-yashiro	50	[3]	(v)	5.33	8 (0, 8)	8	2	Male
2010	Minami-yashiro	51	[3]	(v)	3.71	9 (0, 9)	8	6	Male
2010	Minami-yashiro	53	[3]	(iv)	4.73	10 (0, 10)	7	6	Female
2010	Minami-yashiro	84	[3]	(v)	2.96	8 (0, 8)	8	3	Female
2010	Minami-yashiro	85	[3]	(v)	2.28	4 (0, 4)	4	4	Male
2010	Minami-yashiro	91	[2]	(v)	4.03	7 (0, 7)	3	3	Male
2010	Minami-yashiro	92	[2]	(iv)	1.58	5 (0, 5)	3	1	Male
2010	Minami-yashiro	108	[3]	(iv)	1.74	4 (0, 4)	0	0	Unknown
2015	Kirihata	636	[3]	(v)	3.49	9 (0, 9)	8	5	Male
2015	Kirihata	638	[2]	(iv)	2.66	5 (2, 3)	1	1	Male
2015	Kirihata	642	[2]	(iv)	1.53	8 (0, 8)	0	0	Unknown
2015	Kirihata	644	[2]	(iv)	3.87	7 (2, 5)	5	5	Male

* Developmental stage of brood: [2] = hatchlings, [3] = Middle-sized larvae.

** Nesting phase: (iv) = Closing phase, (v) = Completed.

*** sex of brood could not be determined due to failure of keeping the emerging adults

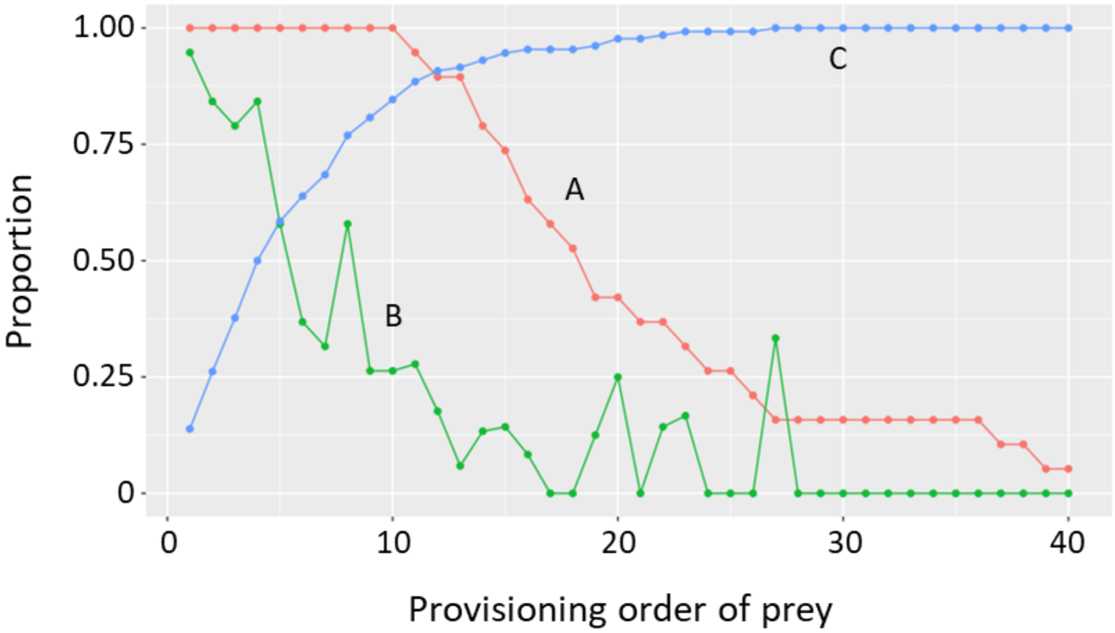

Supplementary Figure S1. Egg-laying pattern in relation to provisioning order of prey observed in 19 nests of *Isodontia harmandi*. A (red curve), existence probability of *i*-th prey item. This curve means that all observed nests had ten prey items, but nests containing 11-th prey items were 18/19 or 0.947, and so on. The nest containing more than 40 prey was only one in this dataset. B (green curve), egg-laying probability, i.e., proportion of prey where an egg was laid on *i*-th prey item. In most of the first prey (94.7%) eggs were deposited. Egg-laying probability was rapidly decreased with provisioning order until around 15th prey item, but sporadically continued on more than 20th prey item. C (blue curve), proportion of cumulative egg numbers. This curve shows that most eggs (c.95%) were laid on up to 17th prey item on the whole.

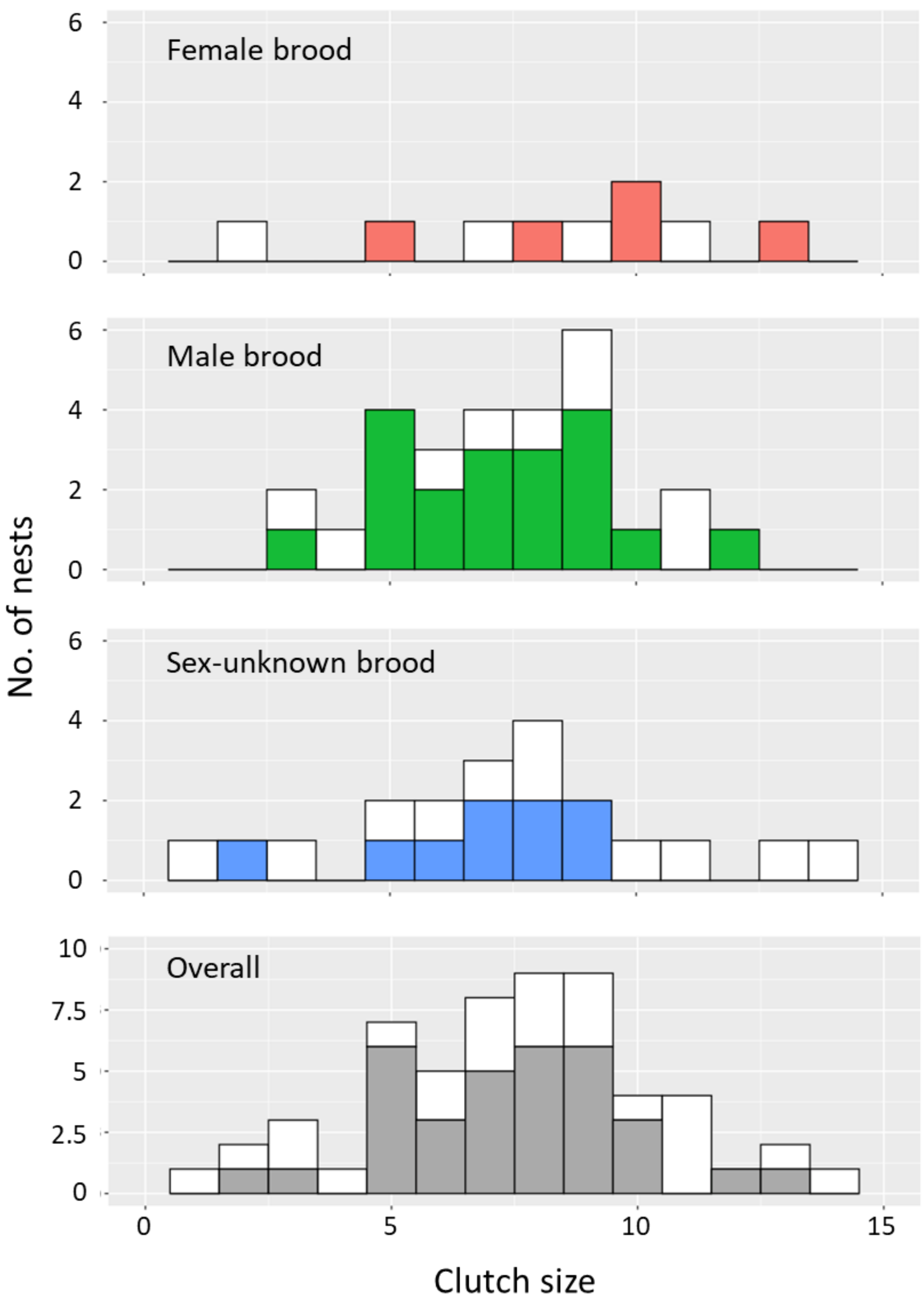

Supplementary Figure S2. Frequency distributions of clutch size in *Isodontia harmandi* nests collected for five years during 2010 and 2015. Data of different years are pooled for female (top), male (upper middle), sex-unknown (lower middle), and all broods (bottom). Filled bars of each sex-category of brood indicate data of the nest where provisioning phase had completed, i.e., their clutch size was absolutely determined. Not-filled bars indicate data of the nest where provisioning phase had not yet completed, but provisioned with more than 17 prey; i.e., their clutch size were approximately estimated.

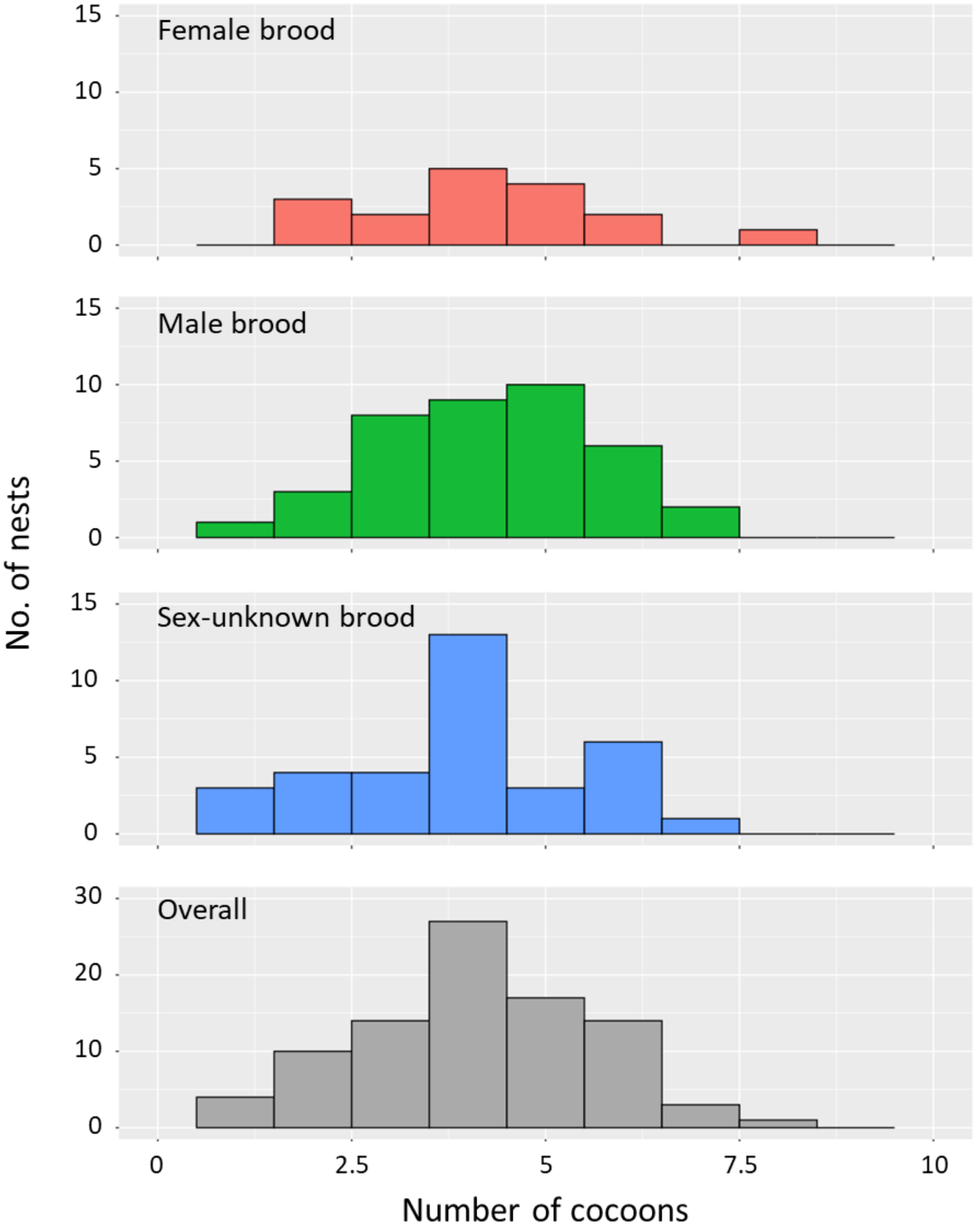

Supplementary Figure S3. Frequency distributions of the number of cocoons produced in *Isodontia harmandi* nests collected for five years during 2010 and 2015. Data of different years are pooled for female (top), male (upper middle), sex-unknown (lower middle), and all broods (bottom).

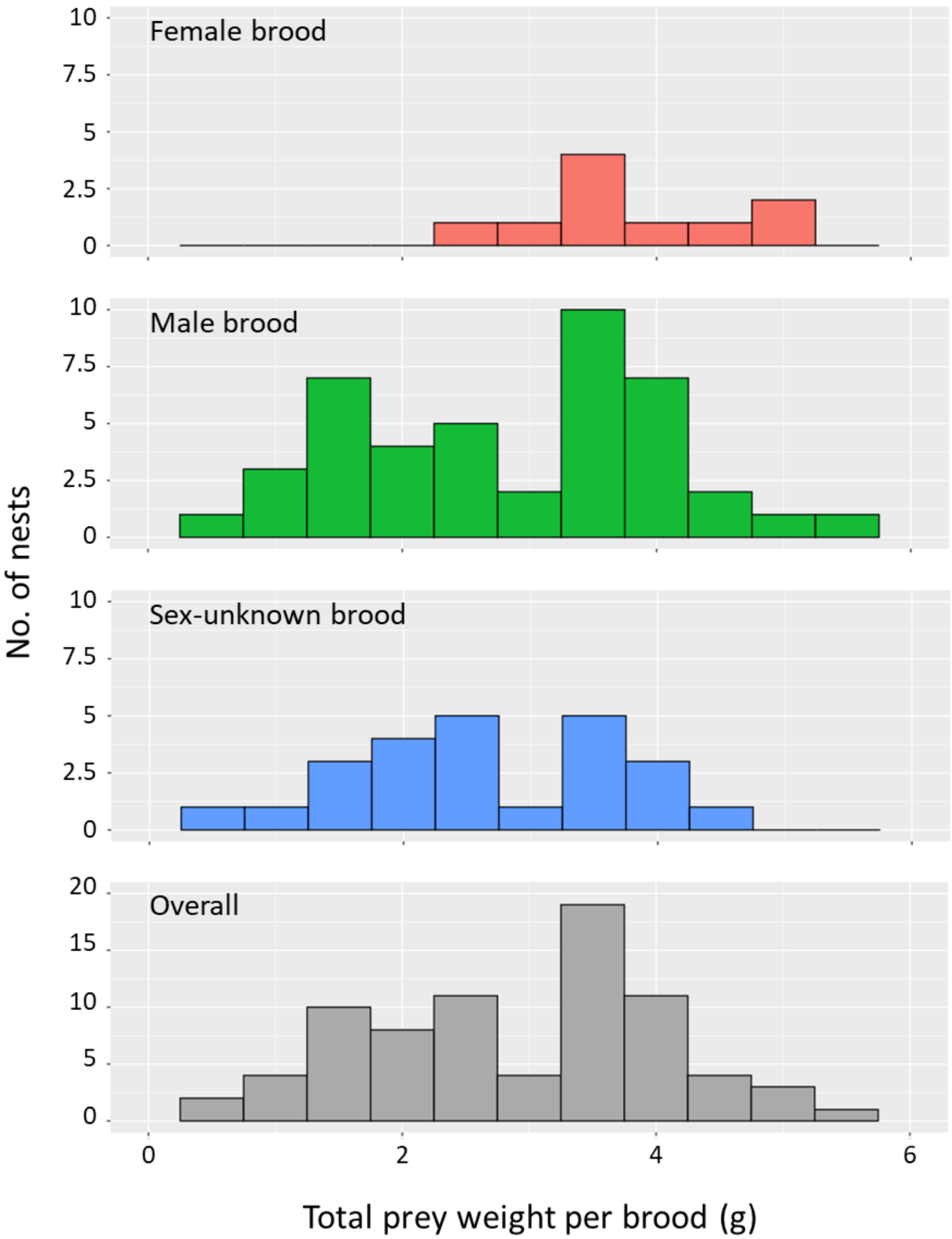

Supplementary Figure S4. Frequency distributions of total prey weight per brood (g) in *Isodontia harmandi* nests collected for five years during 2010 and 2015. Data of different years are pooled for female (top), male (upper middle), sex-unknown (lower middle), and all broods (bottom).

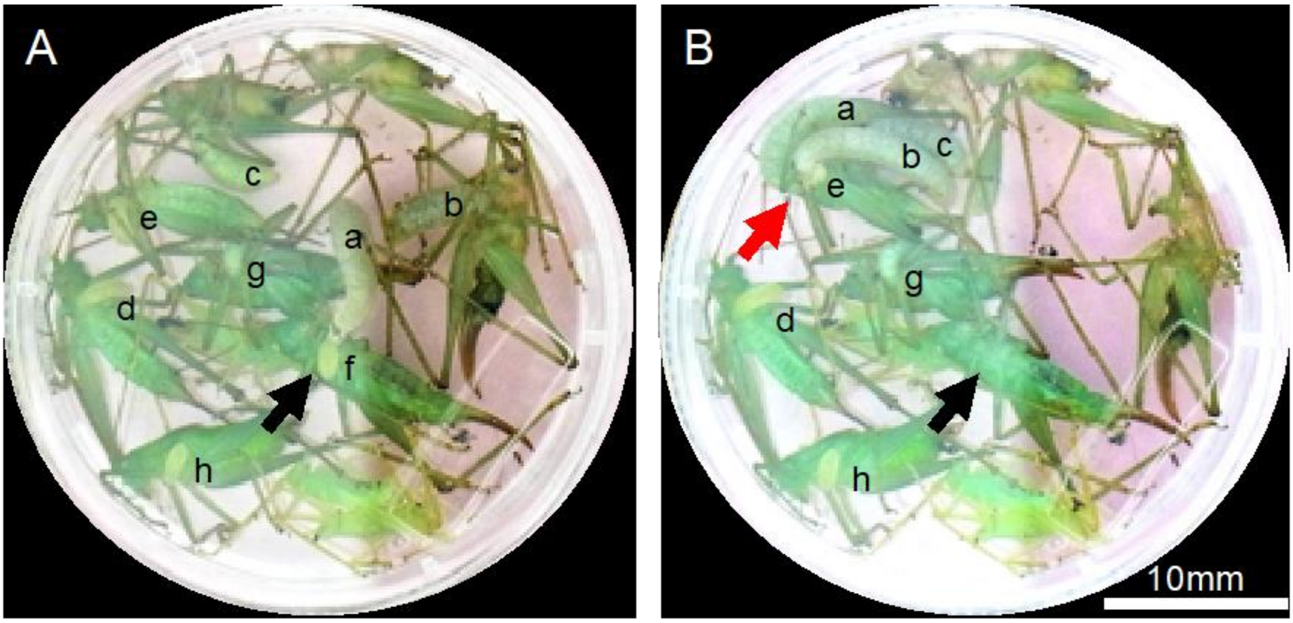

Supplementary Figure S5. Some examples of observations on cannibalistic behavior of larvae in *Isodontia harmandi*. Experiment 2013-1 (see Table1). Initial number of wasp offspring was eight (six eggs and two hatchlings). Experiment started at 17:27 p.m. on July 27, 2013. Around 35 h and 20 mins after the experiment began, the largest larva **a**, which had consumed about half of its own first prey, alighted from the prey and began to move around. Larva **a** touched other wasp larvae and some prey items around itself. About 35 mins later, larva **a** returned to the prey item, which it originally consumed, and soon approached directly the prey item with small wasp hatchling **f** (black arrow in **A**). Larva **a** began to consume hatchling **f** rather than the prey item. This event occurred without any overt aggressive interaction. Consuming hatchling **f** in 30 mins, larva **a** did not try to eat the orthopteran prey to which hatchling **f** had attached (see black arrow in **B**). Around 90 mins after this cannibalistic event, larva **b** (the 2nd largest) started moving around in the Petri dish. Simultaneously, larva **a** and **c** (the 3rd largest) also initiated moving. They got together the prey, which hatchling **e** was feeding. Larva **c** first consumed the prey item and then began to cannibalize the hatchling **e**. Larva **a** joined to cannibalize the hatchling **e** together (red arrow in **B**). Probably larva **b** also shared the victim. It took 33 mins to complete the consumption. After this communal cannibalism, three larvae, **a**, **b**, and **c,** devoured the prey that hatchling **e** was eating.

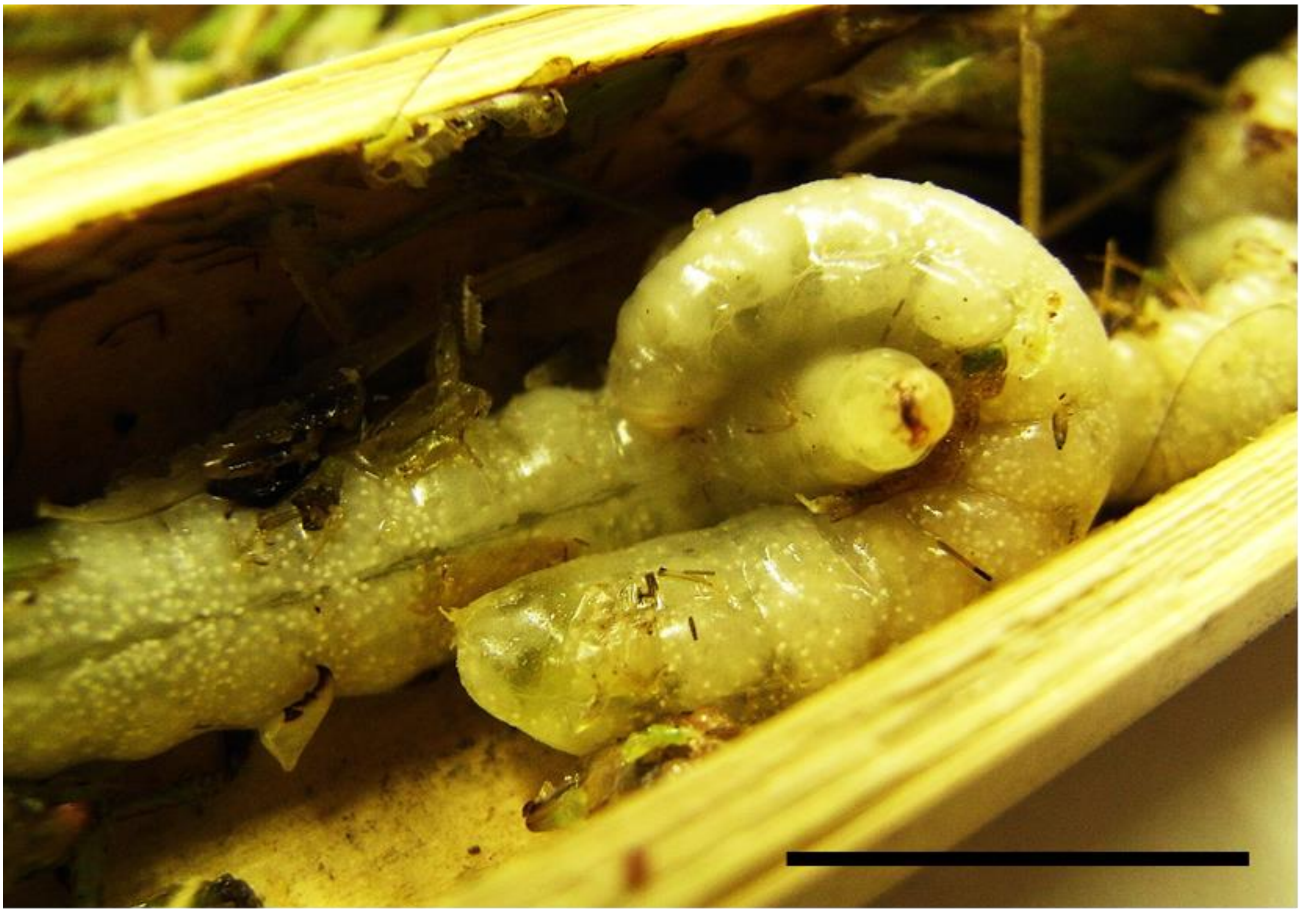

Supplementary Figure S6. Sibling cannibalism in the brood chamber of *Isodonti harmandi*. A larva bending its body like mirror writing of a letter "J" was biting another larva at the thoracic part. Body lengths of both cannibal and its victim were around 20 mm. Scale bar indicates 10 mm at the center of the photo. Photo was taken just after opening the nest built in the bamboo cane trap by YI. Nest was sampled at August 8, 2010. Nest no. 65.

End of Supple.
